# A cell-based drug discovery assay identifies inhibition of cell stress responses as a new approach to treatment of epidermolysis bullosa simplex

**DOI:** 10.1242/jcs.258409

**Published:** 2021-10-13

**Authors:** Tong San Tan, John E. A. Common, John S. Y. Lim, Cedric Badowski, Muhammad Jasrie Firdaus, Steven S. Leonardi, E. Birgitte Lane

**Affiliations:** 1 Skin Research Institute of Singapore, A*STAR, Immunos Building, 8A Biomedical Grove, Singapore 138648; 2 Institute of Medical Biology, A*STAR, Immunos Building, 8A Biomedical Grove, Singapore 138648; 3 A*STAR Microscopy Platform, Immunos Building, 8A Biomedical Grove, Singapore 138648

**Keywords:** Epidermolysis bullosa simplex, EBS, Keratin mutation, EGFR signalling, ERK1/2 signalling, EGFR inhibitors, Pathway intervention therapy drug screen

## Abstract

In the skin fragility disorder epidermolysis bullosa simplex (EBS), mutations in keratin 14 (K14, also known as KRT14) or keratin 5 (K5, also known as KRT5) lead to keratinocyte rupture and skin blistering. Severe forms of EBS are associated with cytoplasmic protein aggregates, with elevated kinase activation of ERK1 and ERK2 (ERK1/2; also known as MAPK3 and MAPK1, respectively), suggesting intrinsic stress caused by misfolded keratin protein. Human keratinocyte EBS reporter cells stably expressing GFP-tagged EBS-mimetic mutant K14 were used to optimize a semi-automated system to quantify the effects of test compounds on keratin aggregates. Screening of a protein kinase inhibitor library identified several candidates that reduced aggregates and impacted on epidermal growth factor receptor (EGFR) signalling. EGF ligand exposure induced keratin aggregates in EBS reporter keratinocytes, which was reversible by EGFR inhibition. EBS keratinocytes treated with a known EGFR inhibitor, afatinib, were driven out of activation and towards quiescence with minimal cell death. Aggregate reduction was accompanied by denser keratin filament networks with enhanced intercellular cohesion and resilience, which when extrapolated to a whole tissue context would predict reduced epidermal fragility in EBS patients. This assay system provides a powerful tool for discovery and development of new pathway intervention therapeutic avenues for EBS.

## Abstract (precis)

**Summary:** High-content screening of an epidermolysis bullosa simplex cell model identifies inhibition of EGFR signalling as a mechanism that can reduce mutant keratin aggregate formation and improve intercellular cohesion.

## INTRODUCTION

Epidermolysis bullosa simplex (EBS) is a rare skin condition usually caused by mutations in the keratin intermediate filament proteins keratin 5 (K5, also known as KRT5) or keratin 14 (K14, also known as KRT14) (Bonifas et al., 1991; Coulombe et al., 1991; Lane et al., 1992), leading to skin blistering and wound development. There is currently no effective treatment beyond palliative care to improve patients' lives. Keratinocytes with the keratin mutations found in severe EBS patients [generalized severe EBS (EBS-gs), also called EBS Dowling–Meara], are under intrinsic stress, mediated by constitutive mitogen-activated protein kinase (MAPK) pathways through JNK1 and JNK2 (JNK1/2, also known as MAPK8 and MAPK9, respectively), p38 proteins, and ERK1 and ERK2 (ERK1/2, also known as MAPK3 and MAPK1, respectively) (D'Alessandro et al., 2002; Morley et al., 2003; Liovic et al., 2008; Russell et al., 2010; Chamcheu et al., 2011a; Wagner et al., 2013). This stress has the features of a misfolded protein response (Chamcheu et al., 2009; Russell et al., 2010), and protein aggregates formed by the mutant keratin are hallmarks of severe EBS (Anton-Lamprecht and Schnyder, 1982). The constitutively active MAPK signalling pathways show stronger and more persistent activation upon additional experimental stress in culture, such as mechanical, heat and osmotic stress, which coincides with increased keratin aggregate formation (Morley et al., 1995, 2003; D'Alessandro et al., 2002; Liovic et al., 2008; Russell et al., 2010; D'Alessandro et al., 2011). This state of stress, as tracked by MAPK activation, is reversible and can be reduced by knocking down the mutant allele using mutation-specific siRNA (Russell et al., 2010), indicating that it is due specifically to the presence of the mutant keratin. The elevated stress in EBS cells has all the hallmarks of priming for wound healing, and indeed EBS cells migrate faster to close scratch wounds than wild-type cells (Morley et al., 2003; Liovic et al., 2009). The desmosomes in EBS cells are also less stable (Russell et al., 2004), as is known to be the case in overtly migratory cells (Wallis et al., 2000), and other junction proteins are expressed at lower levels in EBS cells than in wild-type cells (Liovic et al., 2009). Such profound cytoskeletal differences would render the EBS cells less adhesive to one another, as would be required in wound healing activation.

Additional indicators of stress and wound-like activation of EBS keratinocytes are the prevalent proinflammatory cytokines found to be elevated in blister fluid samples from patients with EBS and other epidermolysis bullosa subtypes (Alexeev et al., 2017). These include EGF and its receptor EGFR, which act in a signalling pathway that is essential for keratinocyte migration and wound closure (Barrandon and Green, 1987; Hudson and McCawley, 1998; Kirfel and Herzog, 2004), and for inducing genes associated with keratinocyte activation to prime for wound healing (Blumenberg, 2013). The presence of these cytokines supports the interpretation of aberrant wound healing and inflammatory responses in EBS-mimetic cells (Lettner et al., 2013; Wally et al., 2013). It has even been proposed that the whole group of epidermolysis bullosa disorders should be considered as systemic inflammatory disorders, rather than skin-limited disorders (Annicchiarico et al., 2015; Esposito et al., 2016).

We hypothesized that reverting the wound activation state of severe EBS (EBS-gs) disease model cells to a more quiescent state should improve the mechanical resilience of the epidermal tissue by increasing cell–cell connectivity and favouring stable filament networks. Building on earlier studies (Law et al., 2009), we have optimized culture conditions for an EBS-gs reporter cell line to achieve maximum consistency and have developed an image-based algorithm using ImageJ for quantification of EBS-gs-mimetic cells with keratin aggregates. This has allowed us to produce a robust assay that is scalable for high-throughput high-content experiments. Screening of a small-molecule library of kinase inhibitors using these EBS-gs reporter cell lines identified a group of compounds all implicated in EGFR signal transduction pathways. Treatment with the FDA-approved EGFR inhibitor afatinib reduced keratin aggregates in the EBS-gs reporter cells and improved the keratin filament network, largely through ERK1/2 inactivation. We show that afatinib treatment drives EBS-gs cells into a quiescent state with increased cell–cell connectivity, junctional proteins and intercellular strength. These findings could have implications for a pathway intervention therapy approach for EBS and possibly for other diseases as well.

## RESULTS

### Optimization of EBS-gs keratinocyte model reporter cell lines – ERK1/2 inactivation reduces keratin aggregates

**Fig. 1.**
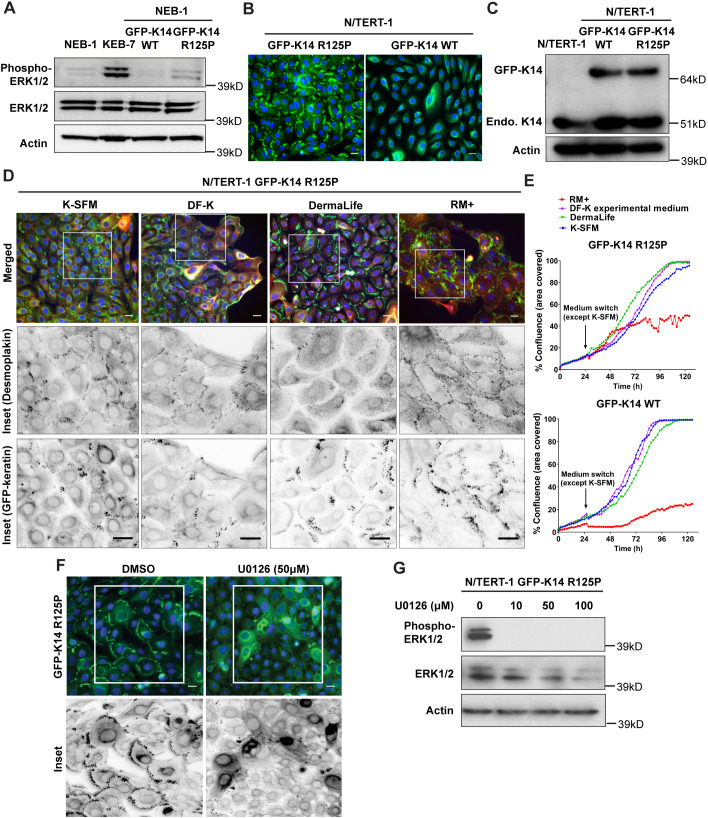
**Optimized EBS-gs reporter cells show reduction of keratin aggregates upon ERK1/2 inactivation.** (A) Immunoblot analysis of phospho-ERK1/2 (Thr202/Tyr204) and total ERK1/2 in cell lysates from wild-type cell lines (NEB-1 and NEB-1 GFP–K14 WT cells) and EBS-gs cell lines (KEB-7 cells, expressing the K14 R125P mutation, and NEB-1 GFP–K14 R125P cells). Actin was used as loading control. kD, kilodaltons. Blots are representative of two independent experiments. (B) Representative fluorescence images of N/TERT-1 GFP-tagged wild-type and GFP-tagged EBS mutant reporter cells stained for nuclei (DAPI, blue). Scale bars: 20 μm. Images are representative of at least three independent experiments. (C) Immunoblot analysis of keratin 14 (LL001) in whole cell lysates from N/TERT-1, N/TERT-1 wild-type and N/TERT-1 EBS mutant cells. Actin was used as loading control. Endo., endogenous. Blots are representative of two independent experiments. (D) Representative fluorescence images of N/TERT-1 EBS mutant cells grown in the indicated cell culture media (K-SFM, DF-K experimental medium, DermaLife and RM+) immunostained for desmoplakin (11-5F) before staining for nuclei (DAPI, blue). In merged images, desmoplakin is in red, GFP–keratin is in green. Boxes highlight regions of interest (ROIs) shown as inverse greyscale single-channel images below. Scale bars: 20 μm. Images are representative of two independent experiments. (E) The proliferation curves of N/TERT-1 EBS mutant (top) and N/TERT-1 wild-type (bottom) keratinocytes were determined using an IncuCyte imaging system, with medium switch from K-SFM to test medium at 24 h post-seeding, monitored until 130 h. Data are taken from one experiment. (F) Representative fluorescence images of N/TERT-1 EBS mutant cells treated with either DMSO or U0126 at 50 μM for 5 h. Boxes highlight ROIs shown in inverse greyscale images below. Scale bars: 20 μm. Images are representative of two independent experiments. (G) Immunoblot analysis of phospho-ERK1/2 (Thr202/Tyr204) and total ERK1/2 in cell lysates from N/TERT-1 EBS mutant cells treated with U0126 at different concentrations (0, 10, 50, 100 μM; drug in DMSO) for 5 h. Actin was used as loading control. Blots are representative of two independent experiments.

Keratinocytes expressing EBS-associated mutations in K14 and K5 can provide valuable models for studying disease mechanisms in tissue culture (Morley et al., 2003). These cells also show the classic hallmark of keratin aggregates (Fig. 1) (Anton-Lamprecht and Schnyder, 1982). NEB-1 keratinocytes engineered to express plasmid-derived GFP–K14 R125P mutant protein to mimic EBS-gs (referred to as NEB-1 EBS mutant cells) also show mutation-related elevation of activated ERK1/2 when compared to NEB-1-based keratinocytes expressing wild-type GFP–K14 protein (GFP–K14 WT) (Fig. 1A), similar to the patient-derived KEB-7 keratinocytes (which harbour the p.R125P mutation in K14) that we reported previously (Morley et al., 2003; Russell et al., 2010).

We observed that not all mutant cells show the same level of keratin aggregates, and thus attempted to define conditions with higher reproducibility for the purposes of drug screening. N/TERT-1 keratinocytes immortalized with human telomerase, an immortalized cell line that has been shown to retain good epidermal differentiation behaviour (Dickson et al., 2000; Smits et al., 2017), showed more consistent behaviour than NEB-1 keratinocytes, and so we generated N/TERT-1 keratinocyte reporter lines stably expressing GFP–K14 R125P (referred to as N/TERT-1 EBS mutant cells) or GFP–K14 WT (referred to as N/TERT-1 wild-type cells). These keratinocytes consistently expressed the GFP-tagged recombinant mutant or wild-type K14 protein throughout numerous passages and at a similar expression level in each cell (Fig. 1B,C). Culture conditions were also important to establish a robust assay system (Zupancic et al., 2012). Comparisons of several commercial media (K-SFM, DF-K experimental medium, DermaLife and RM+) (Table S1) established that K-SFM medium gave the most desirable results, retaining keratin aggregates and desmosome formation in all cells, fulfilling conditions that are ideal for compound screening (Fig. 1D). In contrast, DermaLife-cultured cells had very few desmosomes, and some DF-K experimental medium-cultured cells at the colony edge had poorly defined lamellae, which affected the aggregate count. Cells cultured in K-SFM had similar cell growth profiles as those grown in other cell culture media, except for cells grown in RM+ medium, which showed slower cell growth (Fig. 1E). In these N/TERT-1 EBS mutant optimized reporter cells, levels of phospho-ERK1/2 were also consistently higher than those in wild-type cells, as detected using immunoblotting (Fig. S3B). Inactivating ERK1/2 kinases by blocking upstream MEK1 and MEK2 kinases (MEK1/2; also known as MAP2K1 and MAP2K2, respectively) with U0126 resulted in loss of keratin aggregates in the mutant cells (Fig. 1F,G), as observed previously using patient-derived cells (Russell, 2004). These results suggest that these mutant keratinocytes and their control wild-type keratinocyte counterparts, when grown in K-SFM, constitute a robust system in which to investigate the mechanism of keratin aggregate formation and their relationship to the clinical EBS-gs phenotype.

### Assaying for aggregate-reducing compounds to reverse the EBS phenotype

The GFP-tagged keratin aggregates in all EBS-gs model mutant cells were much brighter than the background of GFP-tagged keratin filaments (Fig. S1A shows NEB-1 EBS cells). We therefore developed an ImageJ algorithm to specifically detect keratin aggregates based on fluorescence intensity, coupled with segmentation analyses to define cell boundaries, producing a final image analysis to accurately quantify the number of cells showing keratin aggregates in a variety of circumstances, including after drug treatments (flowchart shown in Fig. S1B). Regions-of-interest (cells) without keratin aggregates (fluorescence intensity below a defined threshold, outlined in yellow) in mutant or wild-type cells were distinguished from cells with keratin aggregates (intensity above threshold, outlined in magenta) by colour coding, as shown in the segmentation image outputs (Fig. S1C shows N/TERT-1 EBS cells).

**Fig. 2.**
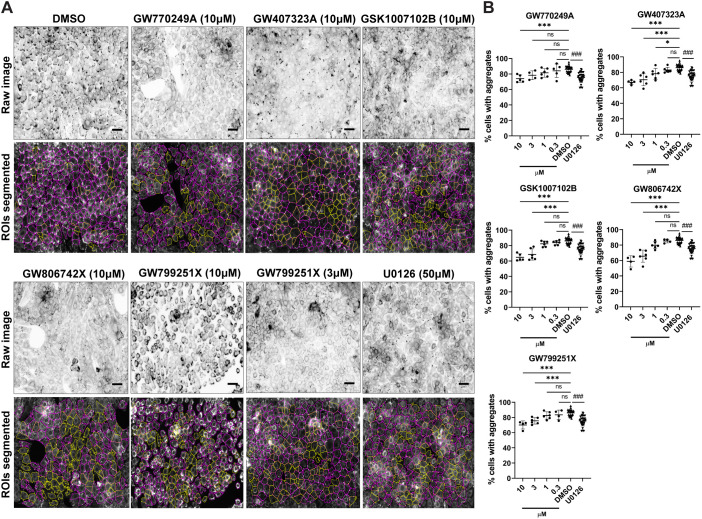
**Screening for compounds from the PKIS library that reverse keratin aggregation in EBS-gs cells.** (A) Representative fluorescence images (inverse greyscale) of N/TERT-1 EBS mutant reporter cells from a secondary screen treated with either DMSO alone or with compounds GW770249A (TIE2/VEGFR2 inhibitor, 10 μM), GW407323A (c-RAF inhibitor, 10 μM), GSK1007102B (AKT1 inhibitor, 10 μM), GW806742X (VEGFR inhibitor, 10 μM), GW799251X (EGFR/ErbB2 inhibitor, 10 μM or 3 μM) or U0126 (50 μM) for 5 h. Cells were stained for nuclei (DAPI, blue). Images were segmented using an ImageJ algorithm for quantification. Magenta and yellow outline regions in the lower panels denote cells with or without keratin aggregates, respectively. ROIs, regions of interest. Scale bars: 40 μm. (B) Quantitative data of a secondary screen of five potential hits at different concentrations (as indicated) using N/TERT-1 EBS mutant cells. Results for potential hits were presented as the percentage of total cells that have aggregates, as mean±s.d. for one to four biological replicates per treatment group in each of two independent experiments. GW770249A (TIE2/VEGFR2 inhibitor), *n*=4–6/group; GW407323A (c-RAF inhibitor), *n*=5–6/group; GSK1007102B (AKT1 inhibitor), *n*=5–6/group; GW806742X (VEGFR inhibitor), *n*=4–6/group; GW799251X (EGFR/ErbB2inhibitor), *n*=4–6/group. DMSO (*n*=37) and U0126 (*n*=30) treatments are negative and experimental reference controls, respectively (same group of DMSO and U0126 treatments plotted in all five graphs), with 14–21 biological replicates in each of two independent experiments. *P<0.05 (*P*=0.02); ***P<0.001 versus DMSO-treated group; ^###^*P*<0.001 versus U0126-treated group; ns, not significant (one-way ANOVA, followed by Tukey's test).

Using the ImageJ algorithm, we screened a library of 352 compounds, the Published Kinase Inhibitor Set (PKIS; Drewry et al., 2014, 2017; Elkins et al., 2016), using N/TERT-1 EBS mutant cells, with DMSO-treated N/TERT-1 EBS mutant cells and N/TERT-1 wild-type cells as negative and positive controls, respectively, as outlined in the workflow procedures (Fig. S2A). The image-based results were analysed, and potential hits were identified based on a cut-off of aggregate reduction of at least 25% (threshold chosen from manual-counting results). Applying this threshold to automated counting identified the same hits as manual counting (Fig. S2B). A secondary screen was performed across further concentration dilutions of the shortlisted hits (Fig. S2C), and this further refined the hits to those showing a dose-response relationship in aggregate reduction. The compounds identified were GW799251X (EGFR/ErbB2 inhibitor), GW770249A (TIE2/VEGFR2 inhibitor), GW407323A (c-RAF inhibitor), GSK1007102B (AKT1 inhibitor), and GW806742X (VEGFR inhibitor) (Fig. 2A,B), with the structures indicated in Fig. S2D. Taken together, these results identified a group of small molecule ‘hits’ that reduced keratin aggregates, most of which are linked to the EGFR signalling pathway.

### Reducing EGF signalling reduces keratin aggregate formation in EBS-gs keratinocytes

**Fig. 3.**
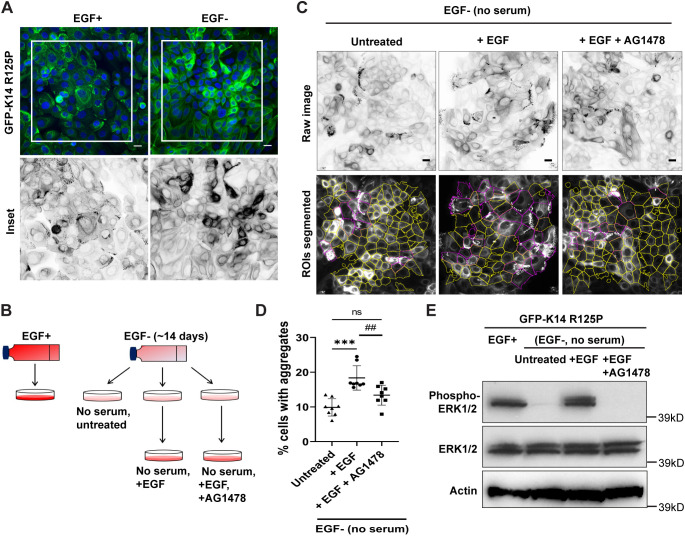
**EGF stimulation increases peripheral keratin aggregates in EBS-gs cells.** (A) Representative fluorescence images of NEB-1 EBS mutant cells cultured routinely in EGF medium (EGF plus, EGF+) and those cultured without EGF (EGF minus, EGF−) medium over ∼14 days. Cells were stained for nuclei (DAPI, blue). Boxes indicate regions shown as inverse greyscale images of GFP fluorescence below. Scale bars: 20 μm. Images are representative of two independent experiments. (B) Schematic diagram of the experimental setups. (C) Representative fluorescence images (inverse greyscale) of serum-starved (no serum/FBS) NEB-1 EBS mutant cells cultured in EGF minus medium (EGF−) that were either untreated or re-stimulated with EGF (100 ng/ml) for 3 h, with or without EGFR inhibitor, AG1478 (10 μM) treatment for 1 h. Cells were stained for nuclei (DAPI, blue). Images were segmented using an ImageJ algorithm for quantification. Magenta and yellow outline regions in the lower panels denote cells with or without keratin aggregates, respectively. ROIs, regions of interests. Scale bars: 20 μm. (D) Quantitative data presented as the percentage of total cells with keratin aggregates, as mean±s.d. for four biological replicates per treatment group in each of two independent experiments. EGF− (untreated), *n*=8; EGF− (+EGF), *n*=8; EGF− (+EGF+AG1478), *n*=8. ***P<0.001 versus untreated group; ^##^*P*<0.01 (*P*=0.009) versus +EGF group; ns, not significant (one-way ANOVA, followed by Tukey's test). (E) Immunoblot analysis of phospho-ERK1/2 (Thr202/Tyr204) and total ERK1/2 in cell lysates from NEB-1 EBS mutant cells cultured normally in EGF plus medium (EGF+), serum-starved (no serum/FBS) cells cultured in EGF minus medium (EGF−) that were either untreated or re-stimulated with EGF (100 ng/ml) for 3 h, with or without EGFR inhibitor AG1478 (10 μM) treatment for 1 h. Actin was used as loading control. kD, kilodaltons. Blots are representative of two independent experiments.

EGF lies upstream of many signalling pathways and drives many different cellular outcomes. Because of the diversity of pathways implicated by the range of identified hits, we focussed on the likely trigger of all these pathways – EGFR activation – to analyse the validity of these observations. We questioned whether EGF could affect keratin aggregate formation in NEB-1 EBS mutant keratinocytes, which are routinely cultured in serum and EGF. Upon examination, it was clear that morphology was affected by the presence of EGF: cells cultured without EGF (EGF minus, EGF−) grew as more tightly apposed cells in compact colonies, with obviously fewer keratin aggregates, than the cells routinely cultured with EGF (EGF plus, EGF+) (Fig. 3A). Serum-starved NEB-1 EBS mutant cells cultured without EGF (EGF minus group) were either left untreated or were incubated in medium supplemented with EGF, with or without the EGFR inhibitor AG1478 (Fig. 3B,C). EGF re-stimulation caused a significant increase in keratin aggregation, which was lessened with AG1478 treatment (Fig. 3D). ERK1/2 activation, through Ras, Raf and MEK1/2, is a major effector arm of the signalling pathway downstream of EGF and its receptor activation. Immunoblot analysis of the phospho-ERK1/2 (Thr202/Tyr204) levels among the different treatment groups showed low levels of phospho-ERK1/2 in the serum-starved EGF minus group (untreated), which increased upon EGF re-stimulation and was abolished upon AG1478 treatment (Fig. 3E). This response was most strongly seen for phosphorylated ERK1 (p44 isoform). These observations demonstrate that inhibition of the EGFR–ERK1/2 signalling pathway is associated, directly or indirectly, with reduced keratin aggregate formation in EBS-mimetic keratinocytes.

### Afatinib treatment reduces keratin aggregation in EBS-gs keratinocytes largely by inhibiting the EGFR–ERK1/2 signalling axis

**Fig. 4.**
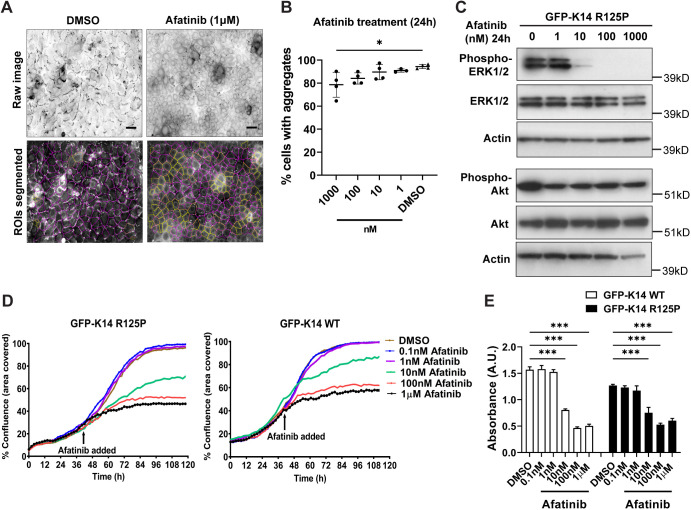
**Afatinib treatment reduces keratin aggregation in EBS-gs keratinocytes largely by inhibiting the EGFR–ERK1/2 signalling axis.** (A) Representative fluorescence images (inverse greyscale) of N/TERT-1 EBS mutant reporter cells treated with either DMSO or afatinib (1 μM) for 24 h. Cells were stained for nuclei (DAPI, blue). Images were segmented using an ImageJ algorithm for quantification. Magenta and yellow outline regions in lower panels denote cells with or without keratin aggregates, respectively. ROIs, regions of interest. Scale bars: 40 μm. (B) Quantitative data from afatinib treatment presented as the percentage of total cells with keratin aggregates, as mean±s.d. for one to two biological replicates per treatment group in each of two to three independent experiments. DMSO, *n*=4; afatinib, *n*=3–4/group. *P<0.05 (*P*=0.02) versus DMSO-treated group (one-way ANOVA, followed by Tukey's test). (C) Immunoblot analysis of phospho-ERK1/2 (Thr202/Tyr204), total ERK1/2, phospho-AKT (Ser473) and total AKT in cell lysates from N/TERT-1 EBS mutant reporter cells treated with afatinib at different concentrations (0, 1, 10, 100, 1000 nM; drug in DMSO) for 24 h. Actin was used as loading control. kD, kilodaltons. Blots are shown from one experiment. (D) The percentage confluence graphs of N/TERT-1 EBS mutant (left) and wild-type (right) cells were determined using an IncuCyte imaging system, with either DMSO or afatinib (0.1 nM to 1 μM) treatments at 42 h post-seeding, monitored until 120 h. Data from one experiment. (E) Cell metabolic/viability (AQ_ueous_ One Solution Cell Proliferation assay) assays to measure the metabolic activities of N/TERT-1 wild-type and N/TERT-1 EBS mutant cells treated with either DMSO or afatinib (0.1 nM to 1 μM) for 72 h. Results presented as mean±s.d. absorbance values in arbitrary units (A.U.) for three biological replicates per treatment group. DMSO, *n*=3/group; afatinib, *n*=3/group. ***P<0.001 against DMSO-treated group (one-way ANOVA, followed by Tukey's test).

Because GW799251X resulted in cell toxicity at 10 µM exposure (Fig. 2A) and at longer incubation times (data not shown), we sought an alternative candidate with lower toxicity, for greater therapeutic suitability. A panel of four FDA-approved EGFR inhibitors (Roskoski, 2019) were tested on the reporter cell system. N/TERT-1-based EBS-gs mimetic keratinocytes were subjected to either afatinib, erlotinib, gefitinib or lapatinib treatment at increasing doses (Fig. 4A; Fig. S3A). Afatinib and lapatinib both reduced keratin aggregates in the reporter cells, but lapatinib required high concentrations, which were clearly toxic to the cells (data not shown); no effect was seen with erlotinib and gefitinib in the tested conditions (Fig. S3C). Only afatinib showed a clear dose-response relationship in reduction of keratin aggregation (Fig. 4B). Hence, we focussed on afatinib in subsequent experiments. ERK1/2 and AKT kinases are the main signalling arms of EGF pathway, and both are implicated in aggregate reduction by the results shown here. Immunoblot analysis of afatinib incubation showed abolition of ERK1/2 phosphorylation (Thr202/Tyr204) at increasing doses (Fig. 4C; Fig. S3B), whereas AKT (Ser473) phosphorylation persisted at these concentrations (Fig. 4C). This suggests that afatinib reduces keratin aggregation in mutant keratinocytes largely through inhibiting the EGFR–ERK1/2 signalling axis.

To assess the broader effects of afatinib treatment, we analysed the cell growth profiles using an IncuCyte imaging system. In both EBS mutant and wild-type N/TERT-1 cells, treatment with increasing doses of afatinib for 72 h showed reduced cell coverage (% confluence) (Fig. 4D). This suggests that afatinib treatments at 10 nM to 1 µM have an effect on cell growth, which agrees well with the afatinib concentrations needed to reduce phosphorylated ERK1/2 kinase levels (Fig. 4C). Cell viability assays also confirmed that the metabolic activity of afatinib-treated cells (10 nM to 1 µM) was reduced when compared to DMSO-treated cells (Fig. 4E).

**Fig. 5.**
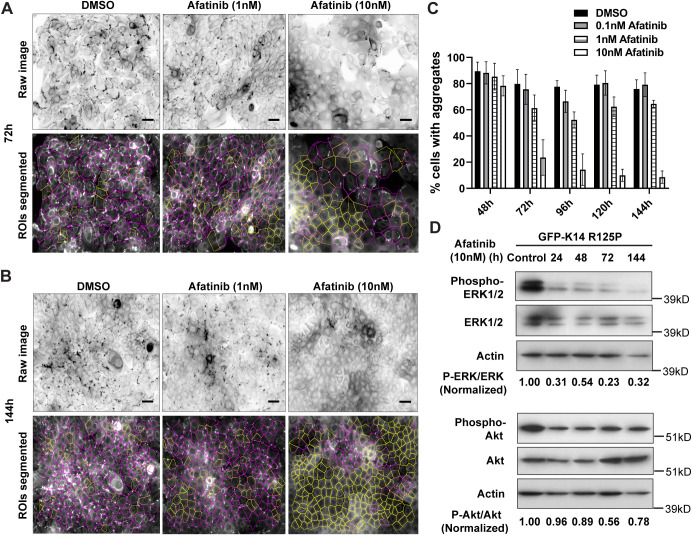
**Titration of afatinib dose and treatment duration to optimally reduce keratin aggregation and its downstream signalling in EBS-gs cells.** (A,B) Representative fluorescence images (inverse greyscale) of N/TERT-1 EBS mutant cells treated with either DMSO or afatinib (1 nM or 10 nM) for 72 h (A) or 144 h (B). Cells were stained for nuclei (DAPI, blue). Images were segmented using an ImageJ algorithm for quantification. Magenta and yellow outline regions in the lower panels denote cells with or without keratin aggregates, respectively. ROIs, regions of interest. Scale bars: 40 μm. (C) Quantitative data from N/TERT-1 EBS mutant cells treated with either DMSO or afatinib (0.1 nM to 10 nM) for 48 h to 144 h, presented as percentage of total cells with keratin aggregates. Mean±s.d. for one to two biological replicates per treatment group in each of two independent experiments. DMSO, *n*=3–4/group; afatinib, *n*=2–4/group. (D) Immunoblot analysis of phospho-ERK1/2 (Thr202/Tyr204), total ERK1/2, phospho-AKT (Ser473) and total AKT in cell lysates from N/TERT-1 EBS mutant cells treated with either DMSO (control) or afatinib (10 nM) for 24 h to 144 h. Actin was used as loading control. Ratios of normalized densitometry values of phospho-ERK1/2 (Thr202/Tyr204):ERK1/2 and phospho-AKT (Ser473):AKT were measured from a single blot experiment using ImageJ. kD, kilodaltons.

Further titration of afatinib exposure over longer times showed that the optimal concentration for keratin aggregate reduction in N/TERT-1 EBS mutant cells was 10 nM at 72 h incubation onwards (Fig. 5A,B). This gave a significant reduction in aggregate-positive cells, with 70–80% of cells having aggregates at the start and ∼10–20% of cells having aggregates after treatment (Fig. 5C). It was found that when the reporter cells were treated with erlotinib, gefitinib and lapatinib for longer periods (48 h and 72 h), concentrations of 100 nM were required to achieve significant keratin aggregate reduction (Fig. S3D,E). Thus, these EGFR inhibitors, with their unique compound structures (Fig. S3F), could all produce the same phenotypic effect, although erlotinib, gefitinib and lapatinib required a much higher concentration than afatinib. The efficacy of afatinib at lower concentrations gives a wider potential therapeutic window, and 10 nM afatinib was used for subsequent experiments. Immunoblot analysis showed a decrease in the levels of phosphorylated forms of ERK1/2 (Thr202/Tyr204) and AKT (Ser473), with the greater effect on phosphorylated ERK1/2 at longer incubation times (Fig. 5D), further supporting the importance of the EGFR–ERK1/2 signalling axis in keratin aggregate formation.

### Afatinib treatment drives proliferating EBS-gs keratinocytes towards quiescence, with minimal cell death over time

**Fig. 6.**
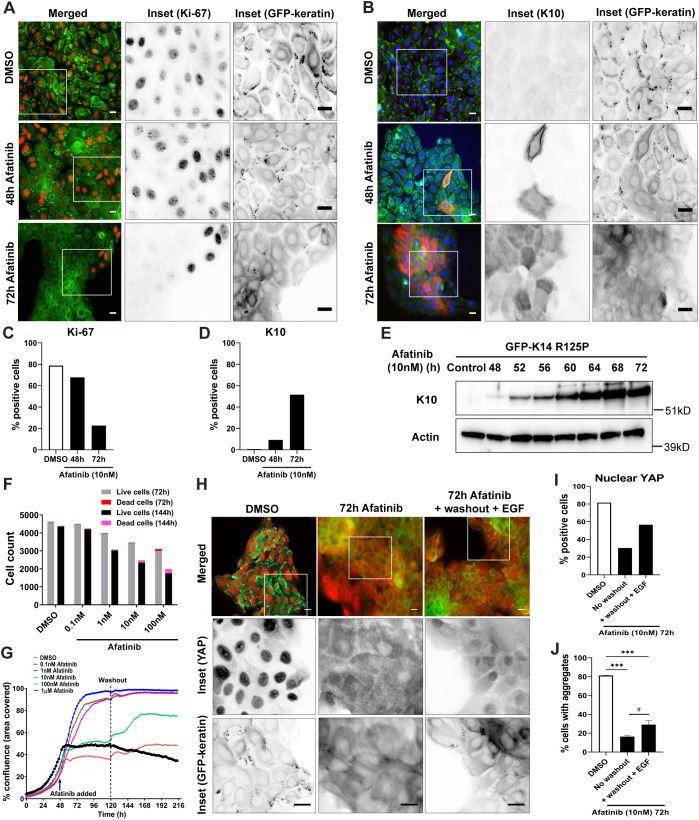
**Afatinib treatment drives proliferating EBS-gs cells towards quiescence, with minimal cell death over time, and proliferative capacity can be restored upon removal of afatinib.** (A,B) Representative fluorescence images of N/TERT-1 EBS mutant cells treated with either DMSO or afatinib (10 nM) for 48 h and 72 h. Cells were immunostained with (A) anti-Ki-67 or (B) anti-K10 antibodies before staining for nuclei (DAPI, blue). Merged images show GFP–keratin in green, and (A) Ki-67 in red and (B) K10 in red. Boxes indicate regions shown as inverse greyscale images on the right. Scale bars: 20 μm. (C,D) Quantitative data of N/TERT-1 EBS mutant cells treated with either DMSO or afatinib (10 nM) for 48 h and 72 h. At least 500 cells were counted in each treatment group (as described in A and B) using ImageJ cell counter plugins, and the results are presented as the percentage of cells positive for either (C) Ki-67 or (D) K10. (E) Western blotting analysis of K10 in whole cell lysates from N/TERT-1 mutant cells treated with either DMSO (control) or afatinib (10 nM) for 48 h to 72 h. Actin was used as loading control. kD, kilodaltons. Blots shown are representative of two independent experiments. (F) LIVE/DEAD assay of N/TERT-1 EBS mutant cells treated with either DMSO or afatinib (0.1 nM to 100 nM) over 72 h or 144 h. Results are presented as the number of cells counted (both live and dead cells) for each treatment group. (G) The percentage confluence graphs of N/TERT-1 EBS mutant cells, as determined using an IncuCyte imaging system, with either DMSO or afatinib (0.1–1000 nM) treatments at 48 h post-seeding and subsequent incubation for 72 h before replacing with fresh medium (washout) and further monitoring until 216 h. Data are representative of two independent experiments. (H) Representative fluorescence images of N/TERT-1 EBS mutant cells treated with either DMSO or afatinib (10 nM) for 72 h, either unchanged or replaced with fresh medium containing EGF (10 ng/ml) for another 6 h (+washout +EGF). Cells were immunostained with anti-YAP antibody before staining for nuclei (DAPI, blue). Boxes indicate regions shown in inverse greyscale images below. Scale bars: 20 μm. (I,J) Quantitative data of N/TERT-1 EBS mutant cells treated with either DMSO or afatinib (10 nM) for 72 h, either unchanged (no washout) or replaced with fresh medium containing EGF (10 ng/ml) for another 6 h (+washout +EGF). (I) At least 500 cells were counted in each treatment group (as described for H) using ImageJ cell counter plugins, and the results are presented as the percentage of cells positive for nuclear YAP. (J) The percentage of total cells with keratin aggregates, as mean±s.d. for two biological replicates per treatment group. DMSO (*n*=2); afatinib-treated, no washout (*n*=2); afatinib-treated, +washout +EGF (*n*=2). ***P<0.001 versus DMSO-treated group; ^#^*P*<0.05 (*P*=0.03) versus afatinib-treated, no washout group (one-way ANOVA, followed by Tukey's test).

Keratinocyte activation is a process that shifts the cells out of quiescence and prepares them for wound healing migration (Freedberg et al., 2001). Previous studies have shown that keratinocytes carrying EBS mutations show many signs of keratinocyte activation and constitutive intrinsic stress (e.g. activated stress kinase pathways, reduced levels of cell junction proteins, increased migration; Morley et al., 2003; Liovic et al., 2008, 2009; Russell et al., 2010; D'Alessandro et al., 2011). EGFR inhibition has been reported to induce a reversible early differentiation state in subconfluent keratinocytes (Peus et al., 1997), which would counteract inappropriate activation. We therefore examined the N/TERT-1 EBS mutant cells for indicators of ‘de-activation’, that is, cessation of proliferation and transition towards differentiation from the start of treatment to the point at which aggregates had disappeared. We used Ki-67 monoclonal antibody (also known as MKI67) as a well-established indicator of cycling cells and keratin 10 (K10, also known as KRT10) as an early differentiation marker to examine the status of mutant keratinocytes over the course of afatinib treatments. Most DMSO-treated mutant keratinocytes at subconfluence showed positive Ki-67 nuclear staining (78.8% of cells Ki-67 positive) and negative K10 cytoplasmic staining (0.6% of cells K10 positive), and this Ki-67 nuclear staining persisted after 48 h afatinib treatment (67.8% Ki-67 positive), although a few K10-positive mutant cells were observed (9.3% K10 positive) (Fig. 6A–D). Upon 72 h afatinib treatment, a reduction in the number of Ki-67-positive cells was seen when most mutant cells have lost keratin aggregates (22.6% Ki-67 positive), with a reciprocal increase in K10-positive cells, especially at the colony centre (51.6% K10 positive) (Fig. 6A–D). These observations suggest that the central part of the colony had become quiescent and receptive to differentiation induction. Immunoblotting analysis confirmed an increase in K10 levels in mutant cells upon afatinib treatment from 48 h to 72 h (Fig. 6E). These results confirm that afatinib-treated keratinocytes become more quiescent and undergo early differentiation.

To confirm that these effects reflected increasing quiescence and not cell death, LIVE/DEAD cell viability assays were performed. These showed less than 4% and 12% cell death in afatinib treatments at tested concentrations over 72 h and 144 h, respectively, in contrast to ∼0.7% cell death in DMSO-treated controls (Fig. 6F; Fig. S3G), indicating that these afatinib-treated cells were still viable in their quiescent state. Removing afatinib after 72 h of treatment led to partial recovery of cell coverage (% confluence) of mutant cells following treatment at 10 nM and 100 nM concentrations (Fig. 6G), but no recovery was seen after treatment at 1 µM, indicating irreversible or persistent EGFR inhibition at this dosage. These results confirmed that the cells were still viable even after longer exposure to afatinib treatment at the tested experimental concentrations.

To further analyse the state of quiescence, YAP (Yes-associated protein, also known as YAP1) distribution was examined. YAP is an effector of the Hippo signalling pathway that translocates to the nucleus when keratinocytes are wound-activated (Lee et al., 2014) but switches to cytoplasmic localization when cells reach confluence and become quiescent (Panciera et al., 2017). YAP subcellular partitioning has recently been shown to be regulated by interactions between K14-dependent disulfide bonding and 14-3-3σ (also known as SFN) in early differentiating keratinocytes (Guo et al., 2020). We therefore used YAP immunostaining to examine the activation status of mutant keratinocytes during afatinib treatment and re-stimulation with EGF. Subconfluent N/TERT-1 mutant cells mostly showed nuclear YAP localization (81.5% nuclear YAP positive), which became more cytoplasmic upon afatinib treatment (30.3% nuclear YAP positive). This indicates that the afatinib-treated mutant cells at the colony centre became quiescent with no keratin aggregates, with the exception of peripheral cells that were still activated, which showed nuclear YAP and some keratin aggregates (Fig. 6H–J). When afatinib was washed out and mutant cells were re-stimulated with EGF, both nuclear YAP (56.6% nuclear YAP positive) and keratin aggregates increased, suggesting an increase in activation of the cells (Fig. 6H–J). Similar observations were also made in afatinib-treated N/TERT-1 wild-type cells (Fig. S4A–E), demonstrating that afatinib inhibition of EGF signal transduction can drive keratinocytes into quiescence. In the EBS-gs cell model, this is accompanied by prevention of the keratin aggregate formation that is the hallmark of severe EBS in patients.

### Afatinib treatment increases levels of junctional proteins and intercellular strength in EBS-gs keratinocyte colonies and monolayers

**Fig. 7.**
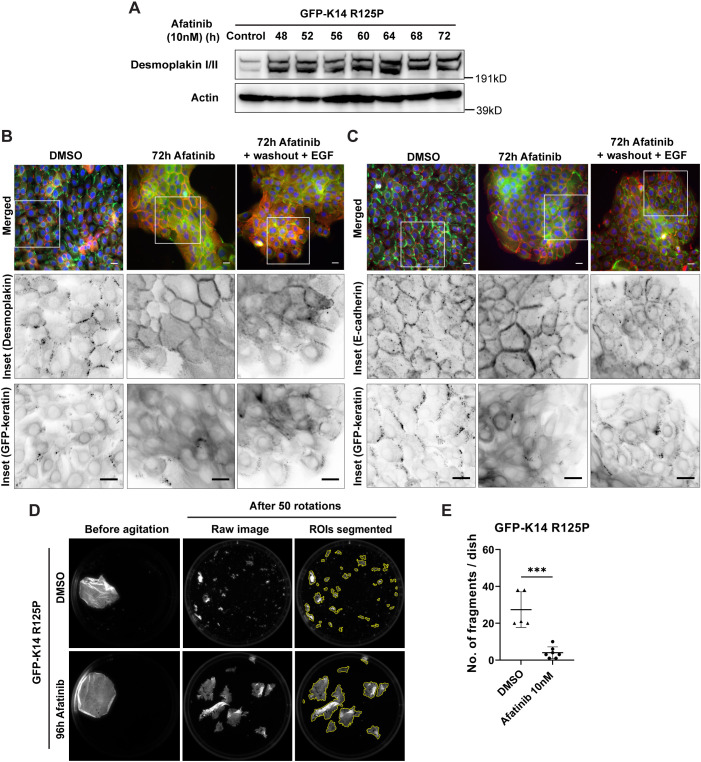
**Afatinib treatment increases junctional proteins in mutant cell colonies and increases intercellular strength in keratinocyte monolayers.** (A) Immunoblot analysis of desmoplakin (11-5F) in whole cell lysates from N/TERT-1 EBS mutant cells treated with either DMSO (control) or afatinib (10 nM) for 48 h to 72 h. Actin was used as loading control. kD, kilodaltons. Blots are representative of two independent experiments. (B,C) Representative fluorescence images of N/TERT-1 EBS mutant reporter cells treated with either DMSO or afatinib (10 nM) for 72 h, either unchanged or replaced with fresh medium containing EGF (10 ng/ml) for another 6 h (+washout +EGF). Cells were immunostained with (B) 11-5F (anti-desmoplakin) antibody or (C) anti-E-cadherin antibody before staining for nuclei (DAPI, blue). Merged images show (B) desmoplakin and (C) E-cadherin both in red, and GFP–keratin in green. Boxes indicate regions shown in inverse greyscale images below. Images are taken from a single experiment. Scale bars: 20 μm. (D) Representative fluorescence images of N/TERT-1 EBS mutant reporter cell sheets treated with either DMSO or afatinib (10 nM) for 96 h, showing fragmentation of the cell monolayer released by dispase incubation after 50 rounds of rotational inversions. Images were segmented using an ImageJ algorithm for quantification. Yellow outline regions denote cell fragments with an area of at least 500 pixels. ROIs, regions of interest. (E) Quantitative data from N/TERT-1 EBS mutant cells treated with either DMSO or afatinib (10 nM) for 96 h, and subsequently agitated as described in D. Data are presented as mean±s.d. number of fragments (≥500 pixels area) for one to six biological replicates per treatment group in each of two independent experiments. DMSO, *n*=5; afatinib, *n*=7. ***P<0.001 against DMSO-treated group (two-tailed unpaired Student's *t*-test).

The loss of keratin aggregates and the denser keratin filament network formation observed as the EBS N/TERT-1 reporter cells became more quiescent would predict a change in cell–cell connectivity in these afatinib-treated EBS mutant cells. We used desmoplakin and E-cadherin (CDH1) as junction protein markers for desmosomes and adherens junctions, respectively, to examine the junctions of mutant keratinocytes over the course of afatinib treatments. Immunoblotting showed an increase in desmoplakin levels in N/TERT-1 EBS mutant cells upon afatinib treatment from 48 h onwards (Fig. 7A). Subconfluent EBS mutant cells showed punctate junctional desmoplakin and E-cadherin staining, which became more intense and continuous in afatinib-treated quiescent cells, suggesting an increase in cell–cell connectivity, which parallelled the loss of keratin aggregates (Fig. 7B,C). When afatinib was removed and mutant cells were re-stimulated with EGF, both desmoplakin and E-cadherin staining, and keratin aggregates, gradually reverted to the subconfluent phenotype seen before drug treatment (Fig. 7B,C).

Finally, to investigate the biological impact of the increased cell–cell junctions, monolayers of afatinib-treated and control N/TERT-1 EBS mutant cells were tested for mechanical resilience of cell–cell adhesion using a dispase-based dissociation assay. The afatinib-treated mutant EBS reporter cells were observed to withstand the mild mechanical stress of repeated rotational inversions much better than control (DMSO-treated) cells (Fig. 7D,E). In this culture system used, similar observations of improved resilience were also seen in afatinib-treated N/TERT-1 wild-type cells (Fig. S5A–E). Thus the afatinib-induced increase in cell connectivity results from decreased keratinocyte activation, or increased quiescence, and is not solely a direct consequence of the presence of mutant keratin.

## DISCUSSION

In this study we investigated whether the keratin aggregates associated with EBS-gs could be used to robustly and safely identify potential therapeutic compounds for EBS. We analysed keratinocytes stably expressing the K14 R125P mutation as a disease model reporter of EBS-gs. In optimized growth conditions, the EBS mutant reporter cells show classical cytoplasmic keratin aggregates, as well as constitutive cell stress by several criteria, including faster migration in closing scratch wounds, and more responsive or constitutively activated stress kinase pathways, as previously described in EBS-gs cell lines (Morley et al., 2003; Liovic et al., 2008, 2009; Russell et al., 2010; D'Alessandro et al., 2011). The presence of keratin aggregates thus parallels the keratinocyte stress ‘activation’ of a wound healing response (Freedberg et al., 2001), during which desmosomes and cell–cell connectivity are reduced and the cytoskeleton is remodelled to prepare the cells for wound healing migration. The formation of the keratin aggregates is likely to be a consequence of wound healing remodelling of keratins, which is disrupted by abnormal assembly kinetics of the mutant keratin, leading to misfolded protein aggregates that trigger cell stress. Thus, these EBS keratin aggregates are potentially useful reporters of wound healing activation and keratinocyte stress status. Assuming that such a partially ‘activated’ state persists in EBS-gs cells in the epidermis *in vivo*, the reinforcing keratin–desmosome network will not be able to operate at maximum strength, which will contribute to the observed tissue fragility under stress. Thus, reversing the keratinocyte activation, by driving these mutant keratinocytes towards quiescence through downregulating ERK1/2 signalling and/or reducing the keratin aggregates (and thus reducing the intrinsic stress), may be a way of reducing the severity of the tissue fragility phenotype of EBS.

We therefore developed a robust drug screening system based on the EBS cell phenotype. The generation and handling of GFP-tagged EBS mutant keratinocytes was optimized to create a highly reproducible experimental cell model system that shows consistent keratin aggregate formation under defined culture conditions. Using these cells, we designed a semi-automated scoring system for measuring reduction of keratin aggregates by image-based computational analysis. Two other studies have described drug screening by counting keratin aggregates as a readout (Kwan et al., 2015; Sun et al., 2016; Spörrer et al., 2019), but these earlier approaches have lower discriminatory potential or lower scalability than the protocol described here. Using the present screening platform, we identified a group of kinase inhibitor compounds related to EGFR signalling that reduce keratin aggregate formation. Keratin aggregates were drastically reduced in EGF-deprived mutant keratinocytes, which could be reversed on EGF re-stimulation. The results described here are all aligned with the concept of EGF as an important extracellular signalling cue that influences keratin remodelling (Windoffer et al., 2011; Felkl et al., 2012; Moch et al., 2013), an essential process for cell migration (Werner et al., 2004). Upon wounding, EGFR signalling is known to trigger changes in keratinocyte morphology, internalization of cell junctions and increased lamellipodia dynamics to facilitate migration to close the wound (Hudson and McCawley, 1998; Hinz et al., 1999; Haase et al., 2003; Chavez et al., 2012).

Activation of the stress kinase ERK1/2 has also been implicated in the elevation of pro-inflammatory thymic stromal lymphopoietin (TSLP) in mouse keratinocytes with EBS-gs mutations, and TSLP is elevated in the serum of severe EBS patients (Kumar et al., 2016). A recent study has also shown that an increased autocrine activation of the amphiregulin (AREG)–EGFR pathway, signalling through Raf–ERK1/2 kinases, induces TSLP expression in keratin-deficient murine keratinocytes, and detectable levels of AREG and TSLP serum proteins have also been found in EBS patients, possibly contributing to itch and inflammation in these patients (Scheffschick et al., 2019). Taken together, our study reinforces the importance of EGFR–ERK1/2 signalling in the pathomechanisms underlying skin fragility and inflammation in EBS.

Reduction of keratin aggregates has previously been used as a readout for therapeutic efficacy of chemical chaperones such as 4-phenylbutyrate and trimethylamine N-oxide (Chamcheu et al., 2009, 2011b; Bchetnia et al., 2016), and anti-inflammatory agents such as diacerein (Wally et al., 2013). Yet reduction of keratin aggregates can only be taken as an indicator of effect per se, not as a conclusive end point, as protein aggregate reduction could also arise from toxic consequences; one of the studies reported that 4-phenylbutyrate impaired cell–cell contacts, cell–matrix adhesion and migration in EBS-gs cells (Spörrer et al., 2019). Where quiescence is a desired end point, and toxicity must be minimized (EBS is a lifelong condition), it will be even more important to distinguish between the cellular health of quiescence and toxic cellular morbidity.

The identification of EGF signalling pathway inhibitors represents a new class of potential therapeutics for EBS, which could be used for pathway intervention therapy, in preference to a more expensive approach of gene correction. Our results support the hypothesis that the constitutively activated EGFR–ERK1/2 signalling in EBS-gs cells primes a ‘wound activation’ state in which cells are less tightly adherent, which would translate into a mechanically more fragile tissue *in vivo*. We show that by inhibiting this pathway, EBS-gs model cells can be driven into a more quiescent state, associated with improved keratin filament networks and cell–cell connections, which could translate into a more mechanically stable epidermis *in vivo* that may resist stress-induced blistering, with clinical benefit to the patients. As afatinib itself has shown adverse side effects on skin in the course of its use for cancer therapy (Lacouture et al., 2013), further work is probably needed to optimize a therapeutic drug, through *in silico* remodelling or identification of downstream signalling targets that elicit the same effects as receptor inhibition. In the search for rare disease therapeutics, more work still needs to be done to understand how we can harness pathway intervention strategies with minimum toxicity.

## MATERIALS AND METHODS

### Antibodies

The following mouse monoclonal antibodies were used as primary antibodies: anti-phospho-ERK1/2 (Thr202/Tyr204) (clone E10, #9106; Cell Signaling Technologies, MA, USA), anti-β-actin (clone AC-15, #A5441; Sigma-Aldrich Corporation, MO, USA), anti-desmoplakin (clone 11-5F; gift from David Garrod, University of Manchester, UK), anti-keratin 14 (clone LL001; Purkis et al., 1990), anti-keratin 10 (clone DE-K10, #M7002; Agilent Technologies, CA, USA), anti-E-cadherin (clone 36, #610182; BD Biosciences, CA, USA) and anti-Ki-67 (clone MM1, #NCL-L-Ki67-MM1; Leica Biosystems, IL, USA). The following rabbit monoclonal or polyclonal antibodies were also used as primary antibodies: anti-phospho-AKT (Ser473) (clone D9E, #4060), anti-AKT (clone C67E7, #4691), anti-phospho-ERK1/2 (Thr202/Tyr204) (clone D13.14.4E, #4370), anti-ERK1/2 (clone 137F5, #4695) and anti-YAP (clone D8H1X, #14074) were purchased from Cell Signaling Technologies (MA, USA). Secondary antibodies used were as follows: goat anti-rabbit IgG (H+L) Alexa Fluor 594 (#A-11037) and goat anti-mouse IgG (H+L) Alexa Fluor 568 (#A-11031) were purchased from Thermo Fisher Scientific (MA, USA), anti-mouse IgG (H+L), HRP conjugate (#W4021) and anti-rabbit IgG (H+L), HRP conjugate (#W4011) were purchased from Promega Corporation (WI, USA).

### Cell culture

KEB-7 cells (K14 R125P mutation) and NEB-1 cells (wild-type) were originally immortalized from patient-derived keratinocytes using HPV16 (Morley et al., 2003). Isogenic pathomimetic cell lines NEB-1 GFP–K14 WT and NEB-1 GFP–K14 R125P were generated previously (Liovic et al., 2009). Cell lines were cultured as described previously (Morley et al., 2003; Liovic et al., 2009), with or without epidermal growth factor (EGF, 10 ng/ml; Sigma-Aldrich Corporation, MO, USA) according to the experimental schedule. N/TERT-1 cells (Dickson et al., 2000) were used to generate reporter lines stably expressing GFP–K14 WT and GFP–K14 R125P, according to previously published methods (Tan, 2012; Tan et al., 2016), with 24 μg/ml of polybrene (Sigma-Aldrich) used to improve infection efficiency. The EBS reporter cells were further sorted using fluorescence-activated cell sorting to ensure that all the cells showed keratin aggregates consistently. These N/TERT-1 cells were routinely cultured in keratinocyte serum-free medium (K-SFM), supplemented with 0.4 mM of CaCl_2_, 0.2 ng/ml of human recombinant EGF and 25 µg/ml BPE (Life Technologies, CA, USA) (Dickson et al., 2000) and used for most experiments. In experiments involving dispase-based dissociation assays, K-SFM medium was substituted with DF-K experimental medium comprising 50% K-SFM and 50% DF-K [DMEM:Ham's F12, 1:1 (v/v), plus BPE (25 µg/ml), L-glutamine (2 mM), EGF (0.2 ng/ml) and 0.3 mM CaCl_2_; Dickson et al., 2000]_._ Cell lines were cultured at 37°C in 5% CO_2_ without fibroblast feeder cells and were routinely checked for mycoplasma infection. See Table S1 for further details of culture media.

### Drug or EGF treatment

Cells were grown on either glass coverslips, dishes or plates prior to DMSO and drug treatments. The MEK inhibitor U0126 (#V1121, Promega Corporation, WI, USA) and the EGFR inhibitors AG1478 (#658552, Calbiochem, MA, USA), lapatinib (#11493, Cayman Chemical, MI, USA), erlotinib (#S7786; Selleckchem, TX, USA), gefitinib (ZD1839; #S1025; Selleckchem, TX, USA) and afatinib (BIBW2992; #S1011; Selleckchem, TX, USA) were all dissolved in DMSO. The structures of afatinib (CHEMBL1173655), erlotinib (CHEMBL553), gefitinib (CHEMBL939) and lapatinib (CHEMBL554) were retrieved from ChEMBL database (https://www.ebi.ac.uk/chembl/). For the EGF study, NEB-1 mutant cells were cultured in medium without EGF (EGF−) for two passages (∼14 days) before the experiments involving serum starvation, EGF re-stimulation for 3 h with or without AG1478 (10 µM) for 1 h. Alternatively, N/TERT-1 wild-type or mutant cells were re-stimulated with EGF for 6 h after afatinib washout.

### The GlaxoSmithKline Published Kinase Inhibitor Set

Cells grown in 96-well black imaging plates (Perkin Elmer, MA, USA) were treated with the GlaxoSmithKline (GSK) PKIS library of compounds (an annotated set of 352 small molecule kinase inhibitors made available to the scientific community (currently Structural Genomics Consortium, University of North Carolina; Drewry et al., 2014, 2017; Elkins et al., 2016) at 10 µM for 4 h. Cells were fixed with 4% paraformaldehyde (PFA), stained with 4-6-diamidino-2-phenyllindole (DAPI) (Sigma-Aldrich Corporation, MA, USA) and washed in phosphate-buffered saline (PBS) before the 96-well plates were imaged using the high throughput ImageXpress Ultra confocal microscope (MDS Analytical Technologies, CA, USA). Structures of PKIS compounds and their targets were retrieved from ChEMBL database (ebi.ac.uk/chembl/). Images were quantified and represented in a 96-well plate format using a macro written with open-source software Fiji (ImageJ) (Schneider et al., 2012). Briefly, this macro is used to load the measurement results into Fiji to generate a colour-coded graphical representation of the input data, allowing clear identification of wells having reduction of keratin aggregates.

### Immunofluorescence microscopy

Cells were grown onto 12 mm^2^ or 22 mm^2^ glass coverslips prior to DMSO and drug treatments. Cells were fixed with either ice-cold methanol and blocked with blocking buffer (3% goat serum in PBS) or fixed with 4% PFA and permeabilized with 0.1% Triton X-100 in blocking buffer, before incubation with primary antibodies overnight. Cells were then incubated with secondary antibodies in the dark, followed by DAPI staining, with PBS washes in between, before being mounted onto slides using Hydromount (Thermo Fisher Scientific, MA, USA) with 2.5% DABCO (Sigma-Aldrich Corporation, MO, USA). Primary antibodies were used at the following dilutions: anti-Ki-67 (1:50); anti-K10 (1:100); anti-YAP (1:100); anti-desmoplakin I/II (1:100); anti-E-cadherin (1:50). Secondary antibodies were used at the following dilutions: goat anti-rabbit IgG (H+L) Alexa Fluor 594 (1:500); goat anti-mouse IgG (H+L) Alexa Fluor 568 (1:500). Images were acquired using the inverted Deltavision epifluorescence microscope and visualized with either an Olympus UApo/340 20× (N.A. 0.75) objective lens; Olympus UApo/340 40× (N.A. 1.35) oil immersion objective lens or an Olympus PlanApo 60× (N.A. 1.42) oil immersion objective lens with a Photometric CCD camera (CoolSNAP HQ2) using the Sedat filter set (Applied Precision, USA). Alternatively, images were acquired using the AxioImager Z1 (Carl Zeiss AG, Germany) with an Axio Cam 506 mono detector, and visualized with a Plan Fluor 40× (N.A. 1.30) oil immersion objective lens. Images were then processed using the softWoRx application (Applied Precision, USA) or ImageJ software. Inversed fluorescence images were presented by displaying greyscale images as black-and-white images using invert LUT in ImageJ software.

### Immunoblotting

Cells were seeded onto 100 mm Petri dishes prior to DMSO and drug treatments. Cells were washed, scraped, collected in ice-cold PBS and centrifuged to obtain cell pellets. The pellets were lysed in 100 µl of freshly prepared lysis buffer containing 20 mM Tris-HCl (pH 7.6), 140 mM NaCl, 5 mM EDTA, 1% (v/v) NP-40 and 0.5% (w/v) sodium deoxycholate, supplemented with a cOmplete™, Mini (EDTA-free) protease inhibitor cocktail tablet (Roche AG, Switzerland) per 10 ml lysis buffer, 1 mM Na_3_VO_4_ and 1 mM PMSF, 5 mM NaF and 50 mM β-glycerolphosphate for 15 min at 4°C. The lysates were separated by centrifugation at 16,000 ***g*** for 15 min at 4°C, and the resulting supernatants were homogenized through a QIAshredder column tube (QIAGEN, Germany). Alternatively, whole cell lysates were obtained by sonicating the cell pellets in lysis buffer three times at 10 s intervals on ice before homogenization. Protein concentration of supernatants or whole cell lysates was determined by BCA assay (Thermo Fisher Scientific, MA, USA), and standardized using BSA. For SDS–PAGE, 20–40 μg of each sample's supernatant or whole cell lysate was mixed with 4× LDS buffer and 10× NuPAGE sample reducing agent (Thermo Fisher Scientific, MA, USA) and incubated for 5 min at 90°C. The proteins were separated on 4–12% Bis-Tris SDS polyacrylamide precast gels (Thermo Fisher Scientific, MA, USA) at 180 V constant for 50 min. For immunoblotting, proteins in the gel were transferred onto nitrocellulose membranes at 100 V constant for 120 min. The membrane was blocked in TBS-T (TRIS-buffered saline with 10% TWEEN-20 detergent) containing 5% milk before overnight incubation with primary antibodies in TBS-T containing 5% BSA at 4°C. The membranes were then incubated with secondary antibodies and washed with TBS-T before visualization by enhanced chemiluminescence (Bio-Rad Laboratories Inc, CA, USA). Protein levels were quantified from images of immunoblots using ImageJ software. Primary antibodies were used at the following dilutions: anti-phospho-ERK1/2 (1:1000); anti-ERK1/2 (1:1000); anti-phospho-AKT (1:1000); anti-AKT (1:1000); anti-β-actin (1:5000); anti-K10 (1:1000); anti-desmoplakin (1:1000); anti-K14 (1:250). Secondary antibodies were used at the following dilutions: anti-mouse IgG (H+L), HRP conjugate (1:1000) and anti-rabbit IgG (H+L), HRP conjugate (1:1000).

### Dispase-based dissociation assays

Cells were seeded in 35 mm dishes and grown to 80% confluence in K-SFM medium before switching to DF-K experimental medium containing either DMSO or afatinib (10 nM) and incubated for another 96 h. After 96 h incubation, cells were washed with PBS and incubated in 2.4 U/ml Dispase II (neutral protease, grade II; Roche AG, Switzerland) for 30 min at 37°C. The detached monolayers were carefully washed with PBS and transferred to 15 ml conical tubes filled with 5 ml PBS. The tubes were attached to a rocker and subjected to 50 inversion cycles. The fragments were then imaged using a Chemidoc™ MP Imager with a green fluorescence filter (Bio-Rad Laboratories, Inc, CA, USA), and quantitated using a batch processing macro written with open-source software Fiji (ImageJ). This macro was used for automatically batch counting the number of fragments based on a minimum size requirement, by first loading the image, identifying the dish and then counting the fragments within the dish.

### Cell growth and viability assays

To assess cell growth, cells were seeded in 24-well plates and cultured at 37°C and 5% CO_2_ in an IncuCyte system (Essen BioScience, MI, USA). Cell coverage (% confluence) was monitored by acquiring fluorescence and phase-contrast images at 10× magnification. Afatinib was added at concentrations from 0.1 nM to 1 µM, and cells were treated for 72 h. For washout experiments, after 72 h afatinib treatments, the medium in each well was replaced with fresh medium, and the cells were further incubated in the IncuCyte to track the cell coverage profile.

For cell viability/metabolism assays, cells seeded in 96-well plates were treated with DMSO or afatinib at concentrations from 0.1 nM to 1 µM for 72 h. After 72 h incubation, cell viability/metabolism assay was performed using a Celltiter 96 AQ_ueous_ One Solution Cell Proliferation Assay kit (Promega, WI, USA) according to manufacturer's instructions. Briefly, 20 µl of Celltiter 96 AQ_ueous_ One Solution Reagent was added into each well, and cells were incubated for 90 min. The absorbance was then recorded in a 96-well plate reader at 490 nm.

For LIVE/DEAD assays, cells seeded in 24-well plates were treated with DMSO or afatinib at concentrations from 0.1 nM to 100 nM for either 72 h or 144 h. After 72 h or 144 h incubation time points, cells were subjected to LIVE/DEAD cytotoxicity assay according to the manufacturer's instructions (Thermo Fisher Scientific, MA, USA). Briefly, the wells were washed once with PBS, before a solution containing 2 μM calcein AM, 1 μM ethidium homodimer-1 (EthD-1) and 1 μM Hoechst 33342 (Thermo Fisher Scientific, MA, USA) in PBS was added into each well and cells were incubated for 45 min in the dark at room temperature. Images in each well were acquired using the Olympus IX-81 high-content screening inverted microscope (Olympus, Japan), and the number of live and dead cells were quantitated using a macro written with open-source software Fiji (ImageJ). Briefly, this macro was written to automatically execute the image processing steps for counting the total number of nuclei stained with Hoechst (blue). Next, the same macro was also used to count the number of nuclei stained with EthD-1 (red). Finally, the number of live cells was derived by subtracting the number of dead cells (red nuclei) from the total number of cells (blue nuclei).

### High-content imaging

Fluorescence images were acquired using a high throughput ImageXpress Ultra confocal microscope with a 40× Plan Fluor objective (MDS Analytical Technologies, CA, USA). Alternatively, fluorescence images were acquired using the Olympus IX-81 high-content screening inverted microscope, attached with a Marzhauser Scan IM (120×100) motorized XY stage controlled by Ludl Mac5000 and a Photometrics CCD camera (CoolSNAP HQ2), with DAPI and EGFP filters and using a 20× Plan Apo objective (Olympus, Japan).

### ImageJ custom algorithm to define cells with keratin aggregate regions

The custom algorithm was developed for ImageJ software (Schneider et al., 2012) to quantify cells with keratin aggregates. Each image has two channels, *nuclei* and *aggregates*. The algorithm first applies an edge filter to reject any images that are too blurred. For the *nuclei* channel, the algorithm duplicates the image, applies a Gaussian blur to smoothen it, applies a threshold (Otsu, 1979), converts it to a binary image, fills holes in the binary image, and watersheds the image to separate and segment the nuclei. A Voronoi diagram is then generated from the segmented nuclei. The algorithm then removes background portions from the Voronoi diagram based on the background in the *aggregates* channel, and also excludes regions cutting the edge of the image. This finalized Voronoi diagram defines the regions of interest (ROI). For the *aggregates* channel, the algorithm duplicates the image, applies difference of Gaussians to enhance contrast, loops through the ROIs to segment aggregates in each region and tabulates the number of regions with or without aggregates, based on intensity and size. Results are calculated by dividing the number of aggregate-positive regions over total regions segmented and multiplying it by 100.

### Statistical analyses

Data analysis was performed by either two-tailed unpaired Student's *t*-test for two groups or one-way ANOVA followed by post hoc Tukey's test for multiple comparisons of at least three groups, using Prism 9.0 (Graphpad Software Inc.). *P* values of <0.05 were considered statistically significant.

## Supplementary Material

Supplementary information

Reviewer comments
