# Supplementary material for: A cell-based drug discovery assay identifies inhibition of cell stress responses as a new approach to treatment of epidermolysis bullosa simplex
Source: J Cell Sci. 2021 Oct 13;134(19):jcs258409. doi: 10.1242/jcs.258409 (PMC8542385; doi:10.1242/jcs.258409)
Supplement: Supplementary information [file joces-134-258409-s1.pdf]

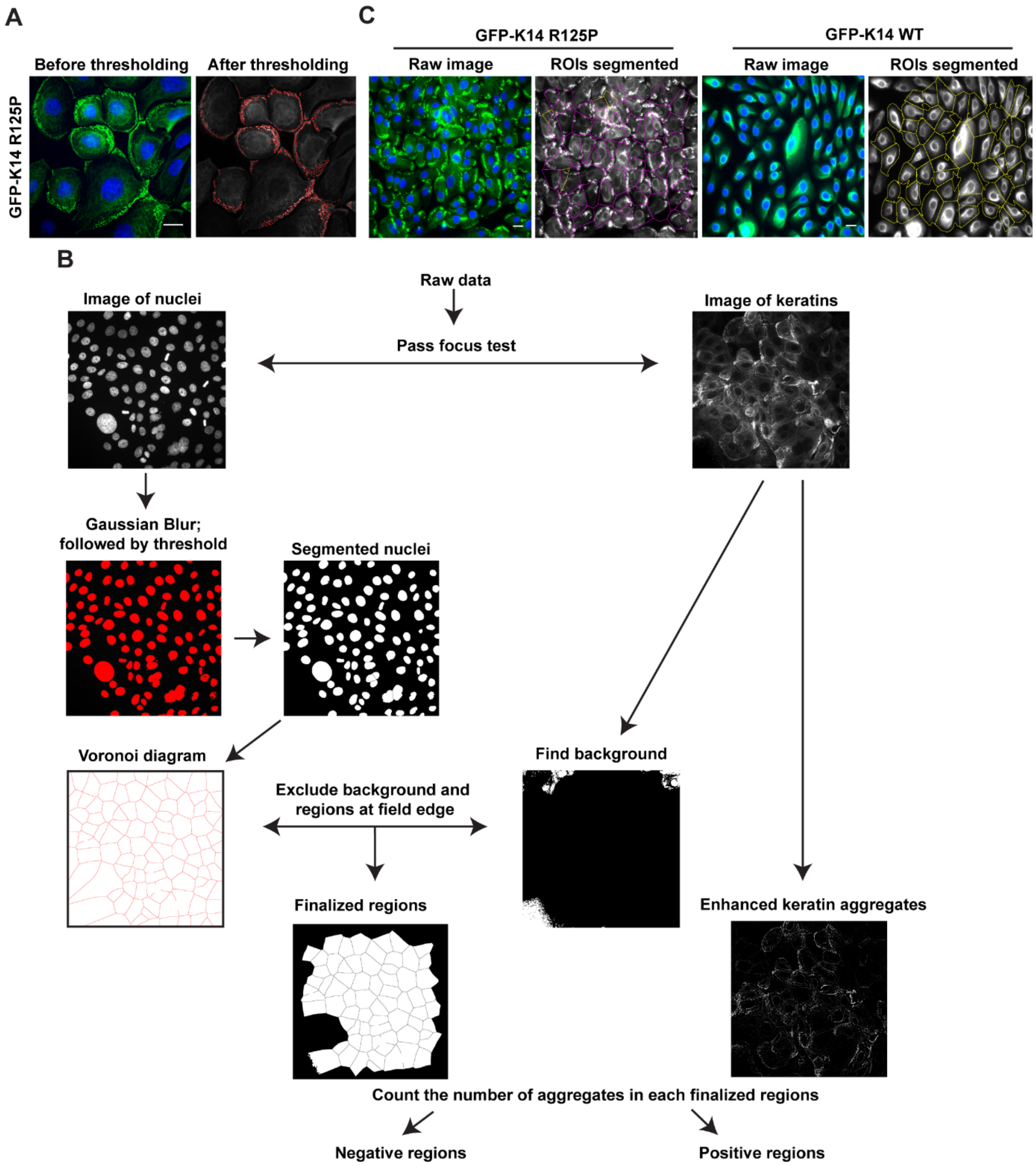

Fig. S1. Semi-automated segmentation strategy to quantify keratin aggregates
(A) Representative fluorescent images of EBS mutant cells stained for nuclei (DAPI, blue), showing peripheral keratin aggregates before and after thresholding (red outlines) in mutant cells using ImageJ software. **(B)** Flow chart with example images of the processing steps in the ImageJ algorithm for counting *aggregates* regions. **(C)** An example of the analysis output of regions of EBS mutant and wildtype cells. Magenta and yellow outline regions denote cells with or without keratin aggregates respectively, based on intensity and size. ROIs: regions-ofinterests. Scale bars: 20 µm.

Journal of Cell Science • Supplementary information

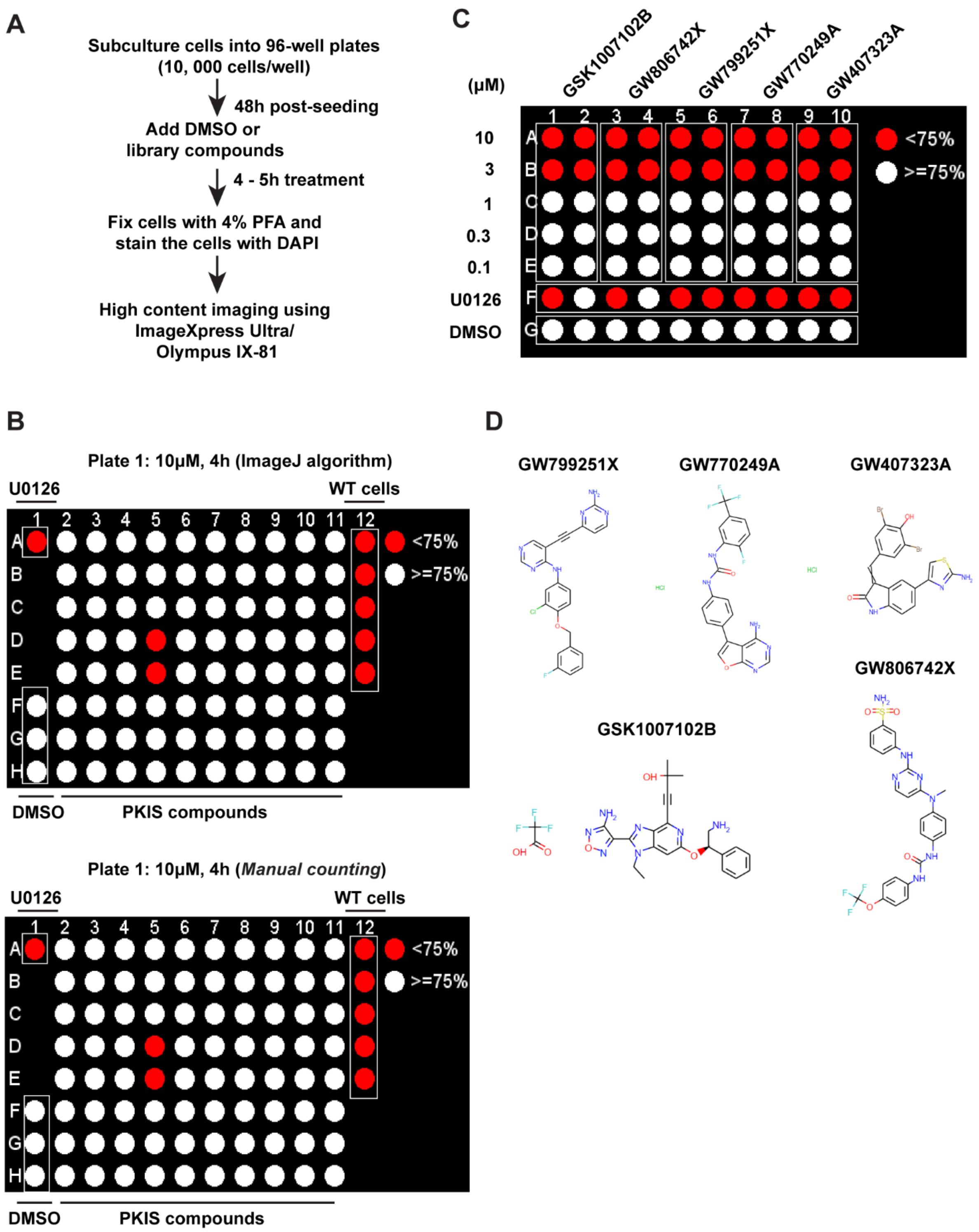

Journal of Cell Science • Supplementary information

Fig. S2. Screening of PKIS library of compounds and its quantification readouts

(A) Workflow of image-based screening of PKIS compounds. **(B)** Preliminary screen of a representative 96-well plate of PKIS library of compounds showing % cells with keratin aggregates quantified either using the ImageJ algorithm or manual counting using the ImageJ cell counter plugins. Red wells: < 75%, White wells: ≥ 75%. WT: wild-type. **(C)** Secondary screening of 5 potential hits: GW770249 (TIE2/VEGFR2 inhibitor), GW407323A (c-RAF inhibitor), GSK1007102B (Akt1 inhibitor), GW806742X (VEGFR inhibitor) or GW799251X (EGFR/ErbB2 inhibitor) arranged and presented as a 96-well plate format showing several titrations from 10 µM to 0.1 µM in duplicates. % of total cells with keratin aggregates were quantified using the ImageJ algorithm. DMSO and U0126 treatments are negative and experimental reference controls respectively. Red wells: < 75%, White wells: ≥ 75%. **(D)** Structures of 5 potential hits (GW799251X/ CHEMBL381604, GW770249A/ CHEMBL588025, GW407323A/ CHEMBL1794053, GSK1007102B/ CHEMBL1909349, GW806742X/ CHEMBL188381) resulting from the secondary screen were retrieved from ChEMBL database (ebi.ac.uk/chembl/).

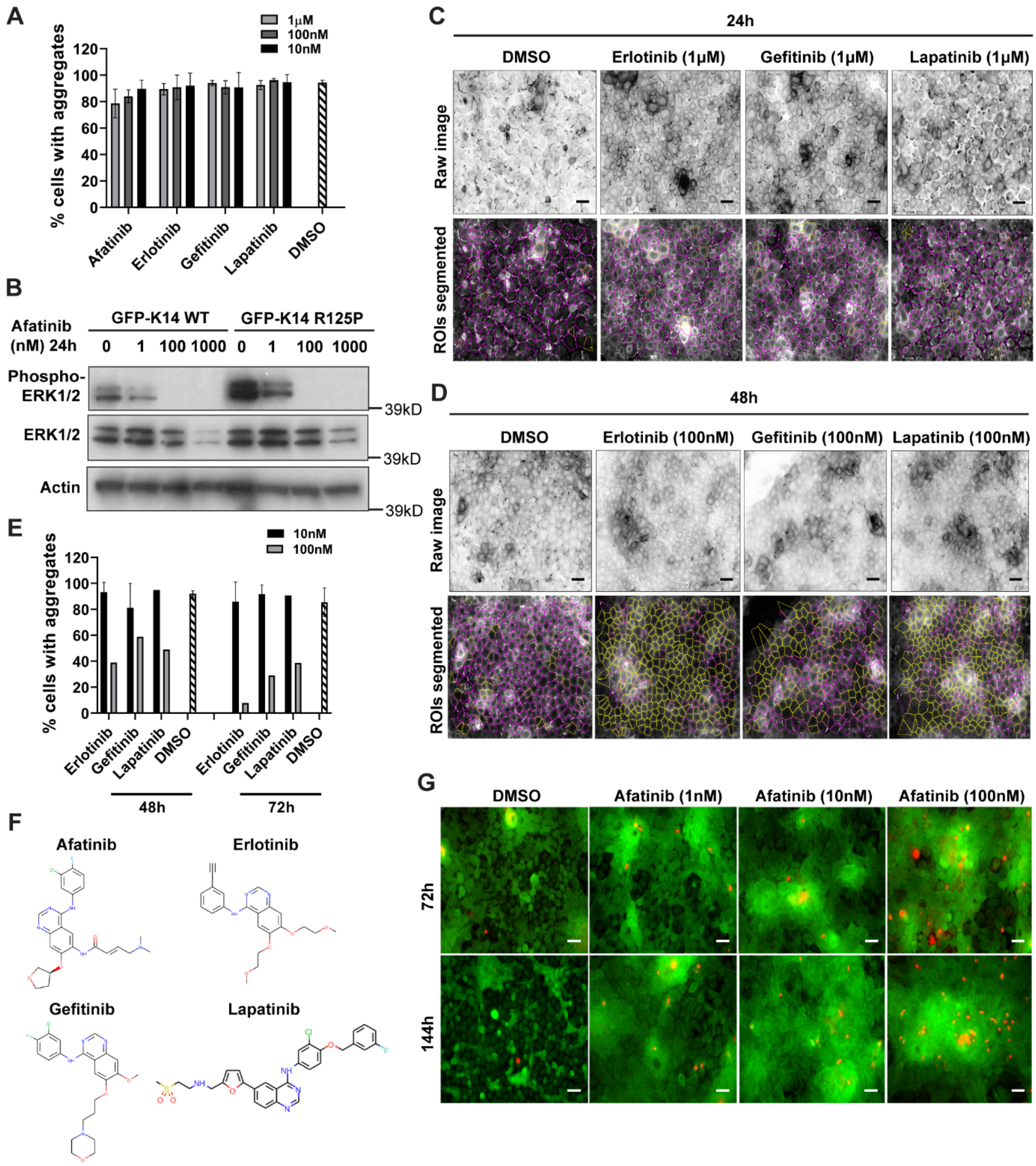

Journal of Cell Science • Supplementary information

Fig. S3. Screening of FDA-approved EGFR inhibitor compounds and EBS-gs cell viability quantification for Afatinib treatment

(A) Quantitative data from a preliminary screen of 4 FDA-approved EGFR inhibitor treatments (Afatinib, Erlotinib, Gefitinib and Lapatinib) at increasing doses using N/TERT-1 EBS mutant reporter cells for 24 h. Results were presented as % of total cells with aggregates, as mean±s.d. for 1-2 biological replicates per treatment group in each of n=2-3 independent experiments. DMSO, negative control. DMSO (n=2), Afatinib (n=4/group), Erlotinib (n=2/group), Gefitinib (n=3-4/group), Lapatinib (n=2-3/group) **(B)** Immunoblot analysis of phospho-ERK1/2 (Thr202/Tyr204) and total ERK1/2 in cell lysates from N/TERT-1 wild-type and EBS mutant cells treated with Afatinib at different concentrations (0, 1, 100, 1000 nM, drug in DMSO) for 24 h. Actin was used as a loading control. **(C, D)** Representative fluorescent images (inverse presentation) of N/TERT-1 EBS mutant cells treated with Erlotinib, Gefitinib or Lapatinib at either 1 µM for 24 h or at 100 nM for 48 h respectively. Cells were stained for nuclei (DAPI, blue). Images were segmented using ImageJ algorithm for quantification. Magenta and yellow outline regions denote cells with and without keratin aggregates respectively. ROIs: regions-of-interests. Scale bar: 40 µm. **(E)** Quantitative data from Erlotinib, Gefitinib and Lapatinib treatments at (10 nM or 100 nM) using N/TERT-1 EBS mutant cells over 48 h or 72 h. Results were presented as % of total cells with aggregates, as mean±s.d. for 1-4 biological replicates per treatment group. DMSO (n=4), Erlotinib (n=1-3/group), Gefitinib (n=1-4/group), Lapatinib (n=1/group) **(F)** Structures of Afatinib (CHEMBL1173655), Erlotinib (CHEMBL553), Gefitinib (CHEMBL939) and Lapatinib (CHEMBL554) were retrieved from ChEMBL database (ebi.ac.uk/chembl/). **(G)** Representative fluorescent images of N/TERT-1 EBS mutant cells treated with either DMSO or Afatinib (1 nM to 100 nM) over 72 h or 144 h. Cells were stained for casein (green), ethidium homodimer-1 (red) and nuclei (Hoechst, blue). Green cytoplasmic staining indicates live cells whereas red nuclei staining indicates dead cells. Scale bars: 40 µm.

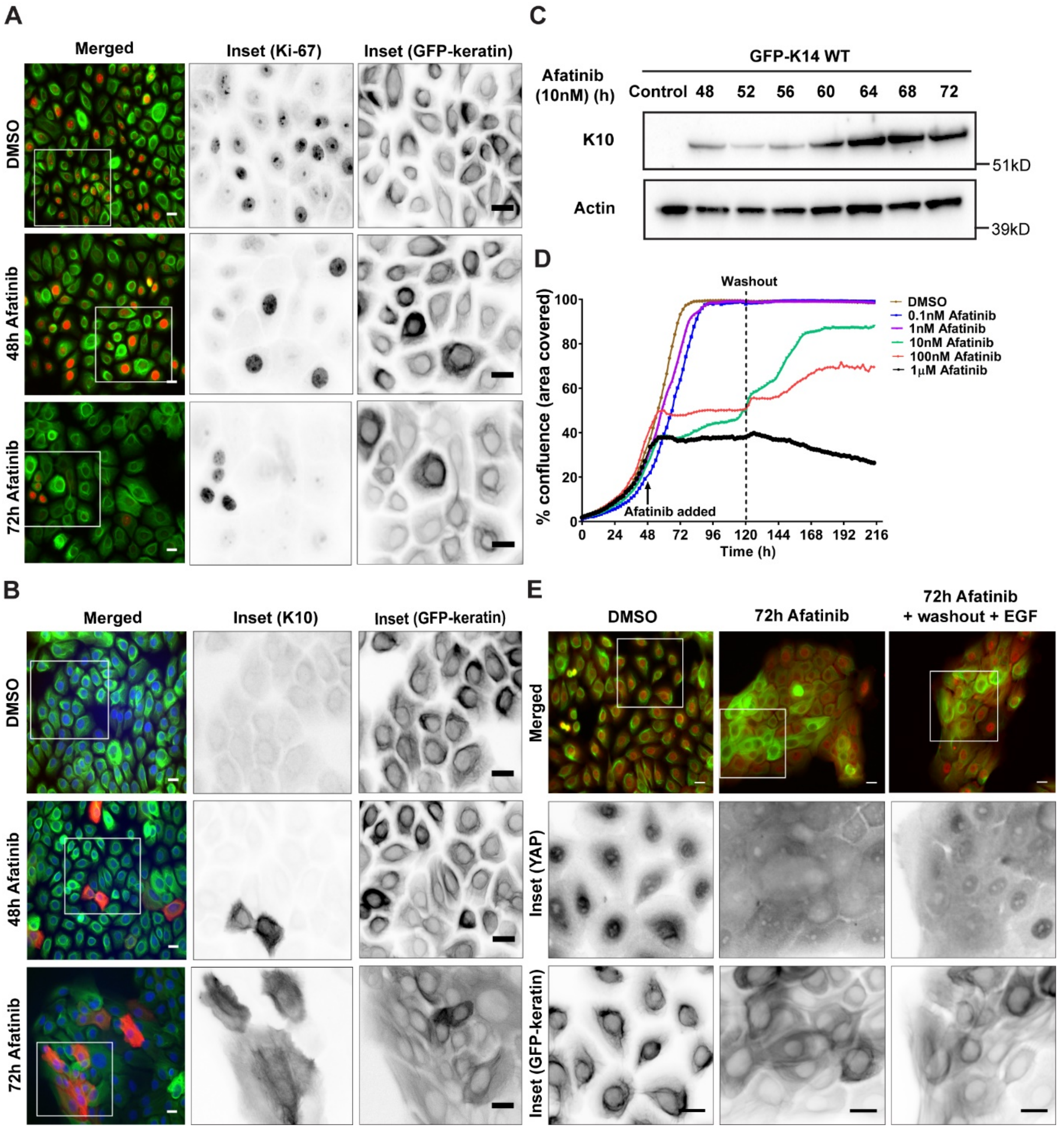

Journal of Cell Science • Supplementary information

Fig. S4. Afatinib treatment drives proliferating wild-type cells towards quiescence, and its proliferative capacity can be restored upon removal of Afatinib

(A, B) Representative fluorescent images of N/TERT-1 wild-type cells treated with either DMSO or Afatinib (10 nM) for 48 h and 72 h. Cells were immunostained with anti-Ki-67 or anti-K10 before staining for nuclei (DAPI, blue). Inset: regions-of-interests (ROIs) demarcated in the white box (inverse presentation). **(C)** Immunoblot analysis of K10 in whole cell lysates from N/TERT-1 wild-type cells treated with either DMSO control or Afatinib (10 nM) for 48 h to 72 h. Actin was used as a loading control. **(D)** The % confluence graphs of N/TERT-1 wild-type cells were determined by the IncuCyte® imaging system, with either DMSO or Afatinib (0.1-1000 nM) treatments at 48 h post-seeding and incubated for 72 h before replacing with fresh medium (washout) and monitored until 216 h timepoint. **(E)** Representative fluorescent images of N/TERT-1 wild-type cells treated with either DMSO or Afatinib (10 nM) for 72 h, either unchanged or replaced with fresh media containing EGF (10 ng/ml) for another 6 h. Cells were immunostained with anti-YAP before staining for nuclei (DAPI, blue). Inset: ROIs demarcated in the white box (inverse presentation). Scale bars: 20 µm.

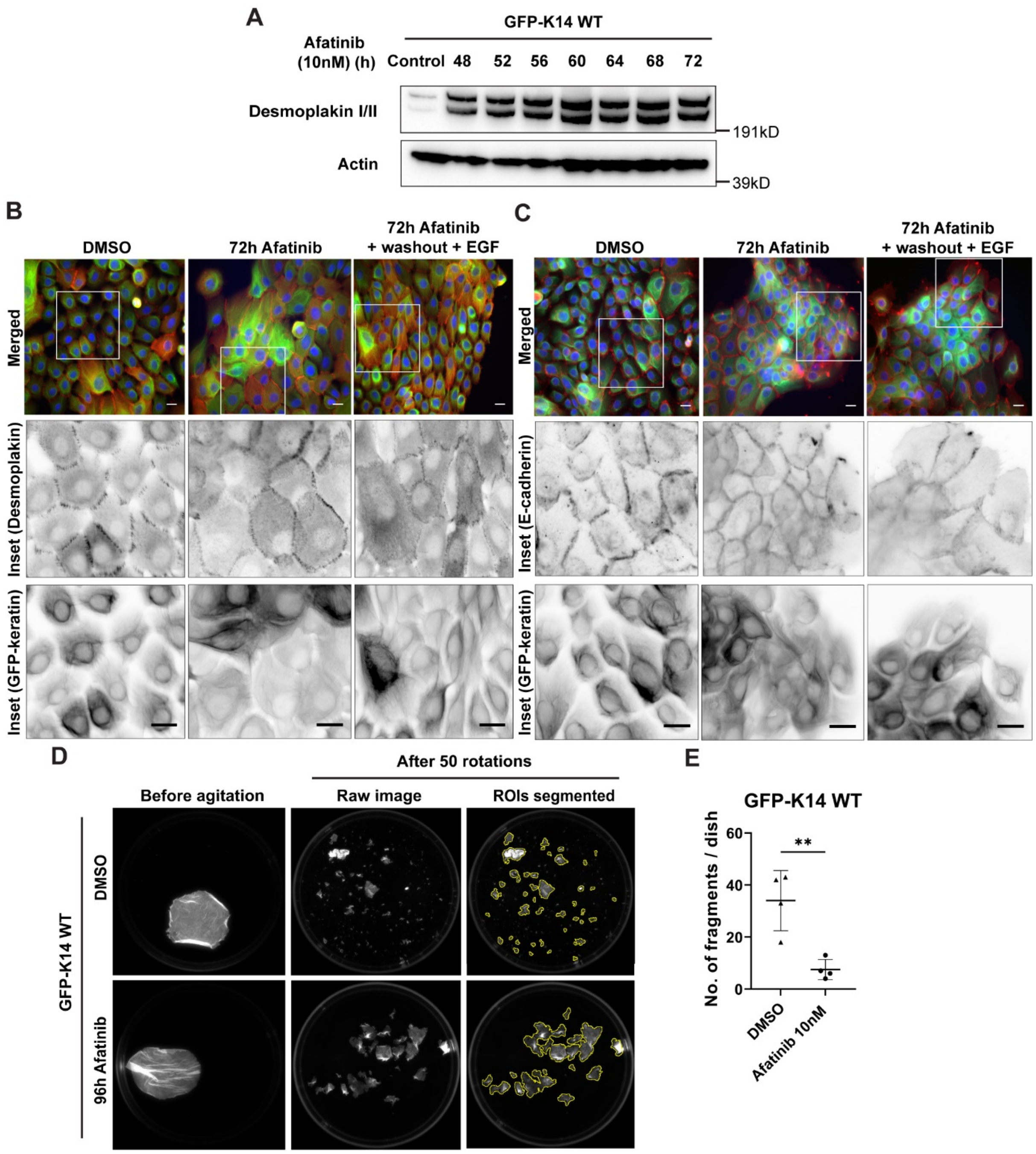

Journal of Cell Science • Supplementary information

Fig. S5. Afatinib treatment increase junctional proteins in wild-type cell colonies and increase intercellular strength in keratinocyte monolayer

(A) Immunoblot analysis of desmoplakin (11-5F) in whole cell lysates from N/TERT-1 wild-type cells treated with either DMSO or Afatinib (10 nM) for 48 h to 72 h. Actin was used as a loading control. **(B, C)** Representative fluorescent images of N/TERT-1 wild-type cells treated with either DMSO or Afatinib (10 nM) for 72 h, either unchanged or replaced with fresh media containing EGF (10 ng/ml) for another 6 h. Cells were immunostained with 11-5F (anti-desmoplakin) or anti-E-cadherin before staining for nuclei (DAPI, blue). Inset: regions-of-interests (ROIs) demarcated in the white box (inverse presentation). **(D)** Representative fluorescent images of N/TERT-1 wild-type cells treated with either DMSO or Afatinib (10 nM) for 96 h, showing fragmentation of the cell monolayer released by dispase incubation after 50 rounds of rotational inversions. Yellow outline regions denote cell fragments with at least 500 pixels area. ROIs: regions-of-interests. **(E)** Quantitative data from N/TERT-1 wild-type cells treated with either DMSO or Afatinib (10 nM) for 96 h presented as average number of fragments (≥ 500 pixels area), as mean±s.d. for 2 biological replicates per treatment group in each of 2 independent experiments. DMSO (n=4), Afatinib (n=4). Statistical analysis was assessed by unpaired student's t-test, ** $p < 0.01$ ($p = 0.005$) against DMSO-treated group. Scale bars: 20 µm.

Journal of Cell Science • Supplementary information

Table S1. Types of medium and its components used
Different types of cell culture medium (K-SFM, D-FK, DF-K experimental medium, DermaLife and RM+) used, its medium components and final concentrations.

Types of medium used	Medium components	Final Concentrations
**Keratinocyte-SFM (K-SFM)**	Bovine pituitary extracts (BPE)[1]	25 µg/ml
	EGF, human recombinant[1]	0.2 ng/ml
	Calcium chloride[2]	0.4mM
**DF-K**	Bovine pituitary extracts (BPE)[1]	25 µg/ml
	EGF, human recombinant[1]	0.2 ng/ml
	Calcium chloride[2]	0.4mM
	DMEM[3] : Ham's F12[1]	1 part: 1 part
	L-glutamine[3]	2mM
**DF-K (Experimental medium)**	K-SFM[1] : DF-K[1]	1 part: 1 part
**DermaLife K**	L-glutamine LifeFactor[4]	6mM
	Extract P™ LifeFactor[4]	0.40%
	Epinephrine LifeFactor[4]	1.0µM
	rh TGF-α LifeFactor[4]	0.5 ng/ml
	Hydrocortisone hemisuccinate LifeFactor[4]	100 ng/ml
	rh insulin LifeFactor[4]	5 µg/ml
	Apo-transferrin LifeFactor[4]	5 µg/ml
**RM+**	DMEM[3] : Ham's F12[1]	3 parts: 1 part
	Hydrocortisone[5]	0.4 µg/ml
	Transferrin[5]	5 µg/ml
	Lyothyronine[5]	2×10^{-11}M
	Adenine[5]	1.9×10^{-4}M
	Insulin[5]	5 µg/ml
	EGF, human recombinant[5]	10 ng/ml
	FBS[1]	10%

Suppliers: [1]Thermofisher Scientific (Massachusetts, USA), [2]Life Technologies (California, USA), [3]GE Healthcare (Illinois, USA), [4]Lifeline Cell Technology (California, USA) and [5]Sigma Aldrich (Missouri, USA).

Journal of Cell Science • Supplementary information
