# Supplementary material for: A cell-based drug discovery assay identifies inhibition of cell stress responses as a new approach to treatment of epidermolysis bullosa simplex
Source: J Cell Sci. 2021 Oct 13;134(19):jcs258409. doi: 10.1242/jcs.258409 (PMC8542385; doi:10.1242/jcs.258409)
Supplement: Reviewer comments [file joces-134-258409_review_history.pdf]

Scalable assay-identified EGFR inhibition lowers stress activation and increases resilience of EBS keratinocytes

Tong San Tan, John E. A. Common, John S. Y. Lim, Cedric Badowski, Muhammad Jasrie Firdaus, Steven S. Leonardi and E. Birgitte Lane

DOI: 10.1242/jcs.258409

Editor: Kathleen Green

Review timeline

Original submission:	15 January 2021
Editorial decision:	3 March 2021
First revision received:	26 July 2021
Accepted:	7 September 2021

Original submission

First decision letter

MS ID#: JOCES/2021/258409

MS TITLE: Epidermolysis bullosa simplex keratinocytes show reduced stress and increased resilience when pushed towards quiescence by EGFR inhibition

AUTHORS: Tong San Tan, John E. A. Common, John S. Y. Lim, Cedric Badowski, Muhammad Jasrie Firdaus, Steven S. Leonardi, and E. Birgitte Lane
ARTICLE TYPE: Research Article

We have now reached a decision on the above manuscript.

To see the reviewers' reports and a copy of this decision letter, please go to: https://submit-jcs.biologists.org and click on the 'Manuscripts with Decisions' queue in the Author Area. (Corresponding author only has access to reviews.)

As you will see, the reviewers are interested in the potential of the work, including the semi-automated work flow, for screening compounds that impact keratin aggregation, but raise a number of substantial criticisms that prevent me from accepting the paper at this stage. The issues raised range from questions about novelty of some of the individual observations, to gaps in the story, to some technical issues. One referee comments that " there is an abundance of unanswered biological questions regarding an EGFR-ERK1/2-K14 aggregation-Mechanical resilience axis that leaves this story feeling rather premature for publication at JCS." Another suggested that the extent to which additional experiments are requested may depend on whether the paper is considered for a regular article or a "Tools and Resources" contribution. Once you've read over all the comments, you may want to consider this latter suggestion as an option, but the choice will be up to you.

If you think that you can deal satisfactorily with the criticisms on revision, I would be pleased to see a revised manuscript. We would then return it to the reviewers.

We are aware that you may be experiencing disruption to the normal running of your lab that makes experimental revisions challenging. If it would be helpful, we encourage you to contact us to discuss your revision in greater detail. Please send us a point-by-point response indicating

© 2021. Published by The Company of Biologists under the terms of the Creative Commons Attribution License (https://creativecommons.org/licenses/by/4.0/).

where you are able to address concerns raised (either experimentally or by changes to the text) and where you will not be able to do so within the normal timeframe of a revision. We will then provide further guidance. Please also note that we are happy to extend revision timeframes as necessary.

Please ensure that you clearly highlight all changes made in the revised manuscript. Please avoid using 'Tracked changes' in Word files as these are lost in PDF conversion.

I should be grateful if you would also provide a point-by-point response detailing how you have dealt with the points raised by the reviewers in the 'Response to Reviewers' box. Please attend to all of the reviewers' comments. If you do not agree with any of their criticisms or suggestions please explain clearly why this is so.

Reviewer 1

Advance summary and potential significance to field

This paper investigated the molecular mechanisms underlying the stress-induced misfolded protein response in Epidermolysis bullosa simplex (EBS), a rare skin condition caused by keratin mutations. The authors developed a semi-automated system to quantify intracellular keratin aggregates using human keratinocytes EBS reporter cells stably expressed GFP-tagged EBS-mimetic mutant keratin. Using this tool, the authors screened a library of kinase inhibitor compounds. They found EGF inhibitor to attenuate mutant K14 protein aggregation formation by downregulating ERK1/2 activation, causing the cells to undergo early differentiation become quiescent, and improve mechanical resilience cell-cell connectivity and formation of stable filament networks. Overall, the paper is well-written. This paper's novelty is the development of a semi-automated ImageJ algorithm to quantify intracellular protein aggregates.

Comments for the author

This paper investigated the molecular mechanisms underlying the stress-induced misfolded protein response in Epidermolysis bullosa simplex (EBS), a rare skin condition caused by keratin mutations. The authors developed a semi-automated system to quantify intracellular keratin aggregates using human keratinocytes EBS reporter cells stably expressed GFP-tagged EBS-mimetic mutant keratin. Using this tool, the authors screened a library of kinase inhibitor compounds. They found EGF inhibitor to attenuate mutant K14 protein aggregation formation by downregulating ERK1/2 activation, causing the cells to undergo early differentiation become quiescent, and improve mechanical resilience cell-cell connectivity and formation of stable filament networks. Overall, the paper is well-written. This paper's novelty is the development of a semi-automated ImageJ algorithm to quantify intracellular protein aggregates; however, more validation is warranted. This paper has some weaknesses and limitations, as addressed in the following

1. In Fig. 1B, the authors need to provide better representative images for GFP-K14 R125P cells staining for GFP-K14 and phospho-ERK1/2, comparable to those in GFP-K14 WT cells (top panels). Current images look as if the cell images were partially chopped off. There's also a black hollow space next to the nucleus. These images are not publication quality. As a result, the immunoreactivity of phospho-ERK1/2 appears to be higher in the GFP-K14 WT cells comparing to GFP-K14 R125P, which contradicts the results of the western blot in Figure 1A.

2. In Fig 1C, while it does appear that phospho-ERK1/2 co-localizes with K14 mutant aggregates in N/TERT-1 cells, phospho-ERK1/2 is primarily expressed perinuclar and to a lesser extent in the aggregates. The activated ERK1/2 expression pattern appears to be different from in NEB-1 mutant cells (Figure 1B), which is mainly at the leading edges and in the aggregates. How do you explain this discrepancy, especially when the author is making claims about the functional link of ERK1/2 to keratin aggregates?

3. Cells in Figures 2A and 2B do not look the same sizes. Do these compounds affect cell growth or cell morphology? Also, it would be more informative to include quantification of aggregates (as in Fig 2C) for all compounds in showed in Fig 2A and immunocytochemistry images in

© 2021. Published by The Company of Biologists under the terms of the Creative Commons Attribution License (https://creativecommons.org/licenses/by/4.0/).

this paper. Overall, showing quantitative analysis with a graphical presentation is more informative than just outlining the images' ROI.

4. How the Image J algorithm works to accommodate a change in cell size and morphology is not clear. The authors need another cell culture model to validate the imaging algorithm since treatment with EGFR inhibitor affects cell morphology and growth.

5. Figure S3D- it's not clear why the authors chose to pursue with EGFR inhibitor when VEGFR inhibitor (GW806742X) and AKT inhibitor (GW1007102B) showed even greater inhibition against mutant aggregates formation.

6. To demonstrate that EGF stimulation caused a significant increase in keratin aggregation via increase ERK1/2 activation, it is important to demonstrate/eliminate its parallel effect on PI3K signaling pathway. It is also recommended that when study phosphorylation of protein using western blot, a phosphatase inhibitor cocktail should be added in the cell lysis buffer during protein extraction to minimize the effect of phosphatases on reducing phosphorylation signals. Not adding phosphatase inhibitors can quickly abolish phosphorylation signals during western blots.

7. Fig 4D showed a different growth rate between WT and mutant cells at Afatinib concentration of 10nM; however, in Fig 4E, cell viability assay doesn't seem to differ at this inhibitor concentration. How do you explain this inconsistency?

8. In Figure 5D, the phosphorylation level of ERK1/2 looks the same between 24 and 72 hr of 10nM Afatinib. If activation of ERK1/2 directly affects mutant aggregates formation, why didn't we see the same effect at 24 hours? Alternatively, if aggregate formation depends on ERK1/2 activation, the level of phospho-ERK1/2 should be significantly different between 24 and 72 hours, but they are not. How do you explain this? Also quantification of western blots is warranted for better illustration of data and reproducibility.

9. Figure 6D, without proper quantification, the immunofluorescence images are not informative at all. The quality of these images was also relatively low, and hard to assess any differences if any.

10. Fig 6F-it's hard to assess changes in the number of aggregate formation between 72 hr Afatinib and after washout with +EGF at this magnification (same for Figure 7B, 7C). It would be helpful to include higher magnification of images and quantification either with the imageJ algorithm or count manually. Also, the GFP signal in 72 Afatinib was overexposed, the quality of the images is low.

11. Fig7A-quality of the desmoplakin western blot was relatively poor compared to the western blot for the same marker in FigS6A.

Minor edit:
1. "The image-based results were analyzed, and potential hits were identified based on a cut-off of 25% aggregate reduction (from manual counting results), which showed similar readouts when compared against manual counting (Fig. S3B)." -Should be against semi-automated counting?

<u>Reviewer 2</u>

Advance summary and potential significance to field

EBS keratinocytes, particularly those from severe forms of the disease, are known to exhibit a wound activated phenotype with cells displaying elevated MAPK signaling, faster migratory capability, and unstable desmosomes that reduce the ability to withstand mechanical stress. The authors hypothesize that a reversion of this wound-like state would render the EBS keratinocytes more mechanically resilient. Using cell lines expressing an EBS-causing mutant K14, the authors performed a kinase inhibitor screen and utilized an ImageJ-based workflow to identify MAPK signaling as a key pathway associated with K14 aggregation. The authors demonstrate that keratin aggregation is induced by EGF exposure and suppressed by EGFR blockade, and that there is an

© 2021. Published by The Company of Biologists under the terms of the Creative Commons Attribution License (https://creativecommons.org/licenses/by/4.0/).

EGFR-K14 aggregate connection that can be manipulated to improve keratinocyte mechanical resilience.

The novelty in the manuscript is with the robust in silico workflow to identify keratin aggregation coupled to the inhibitor screen. Indeed, one can certainly envision this being a powerful screening platform that is scalable to examine libraries of varying scopes and sizes. However, there is an abundance of unanswered biological questions regarding an EGFR-ERK1/2-K14 aggregation-Mechanical resilience axis that leaves this story feeling rather premature for publication at JCS.

Additionally, much of the data presented and the concepts behind their conclusions are already reported in the literature. For instance, EBS cells (particularly those from severe forms of disease) are known to harbor keratin aggregates; mutant K14 expressing cells have reduced intercellular adhesion complexes; mutant K14 expressing cells have elevated levels of activated ERK1/2; EGFR blockade is known to improve intercellular adhesion and mechanical resilience (Liovic, ECR, 2009; Russell, JID, 2010; Klessner MBC, 2009; among many others).

The major finding that appears to be new is that EGFR blockade with low doses of afatanib can reduce mutant keratin aggregation; though, this is clearly not directly related to mechanical resilience since afatinib can also enhance mechanical resilience of cells expressing wild-type keratins.

Comments for the author

Major comments:
1) The level of directness between EGFR inhibition and mutant keratin aggregation is unclear. A number of new data elements would be needed to address this.
a. With the exception of the data using the lower dose afatinib for longer time points, the difference in the % of cells displaying reduced mutant K14 aggregates between conditions (Figure 2C, 3D, S3D, 4B) is rather minimal (roughly from 85% to 70%). Is this level of difference expected to be reflected functionally?
b. Similar to 1a, the EGF stimulated cells only see increase in aggregation from about 10% to maybe 20% of cells. Is this functionally relevant?
c. It seems odd that out of the four drugs used to inhibit EGFR only low dose afatinib seemed to work so well to reduce keratin aggregation. Is there a reasonable explanation for this observation? Afatinib is known to target other ErbB family members, could one of these be involved instead of EGFR?
d. Is there a EGFR specificity prediction for the GW799251X compound?
Could it also target other ErbB family members?
2) How is apoptosis involved in the keratin aggregation/disaggregation?
The authors state that toxicity is occurring, so presumably, the treated cells treated with the compounds are dying off within a day or so after the 10 micromolar treatment. Given that the cells appear to tolerate the lower dose of afatinib for longer periods of time, is apoptosis not triggered in these low dose afatinib treated cells? and could the keratin aggregation/disaggregation observation simply be tied to the apoptotic program more so than to EGFR-ERK activation?
3) Quantification is needed throughout the manuscript for all panels with immunofluorescence images. Without this, one cannot draw a convincing conclusion about the data. For instance, having this data in figure 1 would allow one to make conclusions about the directness of the relationship between ERK activation and mutant keratin aggregation.
a. In figure 1B, what percentages of cells display the border localized phospho-ERK1/2 relative to the peripheral aggregated phospho-ERK1/2?
b. Also in figure 1B, since the authors note there is an uneven expression profile of exogenous K14 forms being expressed, is there any correlation between K14 expression (high versus low) and phospho-ERK1/2 localization?
c. In figure 1C, despite all of the cells in the field expressing mutant K14, only two cells display peripheral aggregation pattern for phospho-ERK1/2. Couldn't one argue then that the majority of mutant K14 expressing cells don't have activated ERK in the peripheral aggregates?

Minor comments:

© 2021. Published by The Company of Biologists under the terms of the Creative Commons Attribution License (https://creativecommons.org/licenses/by/4.0/).

1) It isn't clear why the GW799251X compound was chosen above the shortlisted compounds. They all seem to have fairly similar abilities to reduce the % of cells with keratin aggregates (figure S3D).
2) How do the afatinib concentrations used in the study compare to what is used clinically?
3) The "Common et al., manuscript in preparation" citation in the first paragraph of the Results section is unnecessary. There is no allusion to the significance of non-random aggregate distribution in the manuscript, and there is no way to check the data since it is not accessible to the public.
4) There is a Russell 2016 citation with no information. Is this a searchable source?
5) An observation: the pattern of cells with reduced aggregation appear to be in small clusters in contact with one another (Fig 2, for example).
Might this be an indication of a "seeding" event and/or cell-cell communication that triggers a cascade throughout the field?

Reviewer 3

Advance summary and potential significance to field

Firstly, what is the advance made in the paper and how significant is this for the field?
The submission identifies a strategy to identify small molecules to treat the skin fragility disorder EBS. Using their screening platform the authors identify EGFR inhibitor-based compounds from a library capable of preventing filament aggregation and reducing keratinocyte cell death.
Secondly, do the data reported in the paper justify the conclusions drawn?
Yes, although the evidence of improved biomechanics properties for the treated cells uses an assay system different to others that the group and others have previously developed and published.
Importantly, we ask that referees focus their suggestions for revision on those additions or changes that are necessary for potential acceptance of the manuscript, rather than on potential extensions of the study.
It depends whether this a regular or a technique/methods based submission. In my opinion it fits the latter much better than the former.

Comments for the author

The submission represents an important contribution to our understanding and potential treatment of the skin fragility disease Epidermolysis bullies simplex (EBS). A novel drug screening platform and ImageJ plugin are described and its utility demonstrated by the identification of Afatinib, an EGFR inhibitor and the EGFR signalling pathway in general, to treat EBS.

The experimental system relies on a well-established keratinocyte cell line expressing K14 and the R125P mutant linked to EBS (R125P) and new cell lines based upon N/TERT cells stably transfected with GFP-K14WT and GFP-N14 R125P. This mutation is associated with the Dowling-Meara form of the disease. These KEB7 cells have been examined previously for their biomechanics properties and were shown to be just as capable of surviving large-scale uniaxial strains as cells expressing wild type K14 (Beriault et al 2012), whilst Lulevich et al. (2010) found that KEB7 cells were more compliant and weaker than WT cells when subject to an AFM analysis. It is important to distinguish between apical compression and lateral tension when monitoring the consequences and yet here the group have used a different assay to monitor impact - the resilience of the KEB7 treated cells to a dispase-based dissociation assay. This to me is the weakest part of the submission.

The group had already established that stress kinase pathway, Erk1/2 activation and keratin aggregates are linked. EGF is a known regulator of keratinocyte differentiations and K10 expression. The novelty of this submission is that these different facets have been convincingly connected and set the stage to screen for EGFR signalling pathway inhibitors. The question remains whether this will be effective in animal models and patients, but at least here there is reason to start along that path.
The manuscript is well written, the data presented in a clear and easily tracked manner.

© 2021. Published by The Company of Biologists under the terms of the Creative Commons Attribution License (https://creativecommons.org/licenses/by/4.0/).

First revision

Author response to reviewers' comments

Reviewer 1 Comments for the Author:

This paper investigated the molecular mechanisms underlying the stress-induced misfolded protein response in Epidermolysis bullosa simplex (EBS), a rare skin condition caused by keratin mutations. The authors developed a semi-automated system to quantify intracellular keratin aggregates using human keratinocytes EBS reporter cells stably expressed GFP-tagged EBS-mimetic mutant keratin. Using this tool, the authors screened a library of kinase inhibitor compounds. They found EGF inhibitor to attenuate mutant K14 protein aggregation formation by downregulating ERK1/2 activation, causing the cells to undergo early differentiation, become quiescent, and improve mechanical resilience cell-cell connectivity and formation of stable filament networks. Overall, the paper is well-written. This paper's novelty is the development of a semi-automated ImageJ algorithm to quantify intracellular protein aggregates; however, more validation is warranted. This paper has some weaknesses and limitations, as addressed in the following

1. Reviewer:
In Fig. 1B, the authors need to provide better representative images for GFP-K14 R125P cells staining for GFP-K14 and phospho-ERK1/2, comparable to those in GFP-K14 WT cells (top panels). Current images look as if the cell images were partially chopped off. There's also a black hollow space next to the nucleus. These images are not publication quality. As a result, the immunoreactivity of phospho-ERK1/2 appears to be higher in the GFP-K14 WT cells comparing to GFP-K14 R125P, which contradicts the results of the western blot in Figure 1A.

  Author Response: We agree with the reviewer, thank you for drawing attention to this. We will leave out the immunofluorescence data for pERK as the cell images, although all genuine, are indeed variable and reflect the way keratinocytes can look. (Black hollow space is a vacuole – quite common.) As the data are not essential for the argument, we have removed the confusing ERK immunofluorescence data.

2. Reviewer: In Fig 1C, while it does appear that phospho-ERK1/2 co-localizes with K14 mutant aggregates in N/TERT-1 cells, phospho-ERK1/2 is primarily expressed perinuclar and to a lesser extent in the aggregates. The activated ERK1/2 expression pattern appears to be different from in NEB-1 mutant cells (Figure 1B), which is mainly at the leading edges and in the aggregates. How do you explain this discrepancy, especially when the author is making claims about the functional link of ERK1/2 to keratin aggregates?

  Author response: See response to point 1 above. Because single images cannot reflect the cell variability observed, we agree that this could be misleading and have therefore deleted the ERK immunofluorescence figures from the paper. This also allows us to reorganise the figures and reduce the overall number of items in Supplementary Material by 1, as requested by the editors, by combining Supplementary Fig S1 and Fig 1 into the new Fig 1.

3. Reviewer: Cells in Figures 2A and 2B do not look the same sizes. Do these compounds affect cell growth or cell morphology?

  Author Response: Images were taken at different magniifcations for 2A vs 2B and were collected with different microscopy platforms from 2 different screens. We have now standardized this and show only images from the secondary screening assay, with the same magnification, in the new Fig 2. Some of these compounds, especially GW799251X, do indeed affect cell morphology at higher concentration (10□M), but there is no impact on morphology at the lower concentration (3□M).

Reviewer: Also, it would be more informative to include quantification of aggregates (as in Fig 2C) for all compounds in showed in Fig 2A and immunocytochemistry images in this paper. Overall, showing quantitative analysis with a graphical presentation is more informative than just outlining the images' ROI.

  Author Response: The quantitation (actual cell count in numbers) is already provided in

© 2021. Published by The Company of Biologists under the terms of the Creative Commons Attribution License (https://creativecommons.org/licenses/by/4.0/).

Supplementary Fig S3D. In response to the reviewer's comments, we have now moved it to a more obvious place, in the revised Fig 2, for greater visibility. We have added references to this in the text alongside the relevant figures. We have also now included the actual cell counts for all compounds used.

4. Reviewer: How the Image J algorithm works to accommodate a change in cell size and morphology is not clear. Author Response: This version of the imaging algorithm does not take size and shape parameters of the cells into account. In the experiments described here, this was not necessary because the only parameter being scored here, intentionally, was the number of cells with aggregates (as % of all cells counted), and NOT the number of aggregates per cell, which may vary with different cell size and morphology.

Reviewer: The authors need another cell culture model to validate the imaging algorithm since treatment with EGFR inhibitor affects cell morphology and growth.

Author Response: We agree that it is important to test the algorithm with more than one cell model. Because only keratinocytes and EBS keratins are relevant to this assay, we ran the algorithms with multiple versions of the EBS keratinocyte reporter cells, using the NEB-1 (Fig 3) and the N/TERT-1 based cell lines. All the lines gave cognate results and distinguished clearly between those with EBS mutant keratin 14 and those with wild type K14.

5. Reviewer: Figure S3D- it's not clear why the authors chose to pursue with EGFR inhibitor when VEGFR inhibitor (GW806742X) and AKT inhibitor (GW1007102B) showed even greater inhibition against mutant aggregates formation.

Author Response: All the hit compounds identified in the kinase inhibitor screen, except maybe one, have targets in or affected by the EGF/EGFR signalling pathway. We focused on EGFR for the reason that this is the most upstream target in the pathway and therefore the definitive component in the signaling cascade.

6. Reviewer: To demonstrate that EGF stimulation caused a significant increase in keratin aggregation via increase ERK1/2 activation, it is important to demonstrate/eliminate its parallel effect on PI3K signaling pathway.

Author Response: We agree with the Reviewer's comment and acknowledge that, since EGF stimulation is known to activate PI3K signaling pathway, inhibition of EGF signalling is likely to dampen PI3K activity as well. However inhibition of the MEK/ERK1/2 pathway alone with U0126 appears sufficient to induce a dramatic reduction in mutant keratin aggregation (former Fig 1D, now Fig 1F), suggesting that the involvement of PI3K signaling in this process is likely to be smaller. ERK1/2 activation is one of the main consequences of EGFR activation, and using Afatinib we did show that AKT was less affected. We have moderated the text accordingly to be more circumspect about a sole role for ERK.

Reviewer: It is also recommended that when study phosphorylation of protein using western blot, a phosphatase inhibitor cocktail should be added in the cell lysis buffer during protein extraction to minimize the effect of phosphatases on reducing phosphorylation signals. Not adding phosphatase inhibitors can quickly abolish phosphorylation signals during western blots.

Author Response: We agree with this comment. We have already incorporated phosphatase inhibitors (sodium orthovanadate (Na_3VO_4), sodium fluoride (NaF) and ß-glycerolphosphate) throughout this study, as explained already in the Methods section.

7. Reviewer: Fig 4D showed a different growth rate between WT and mutant cells at Afatinib concentration of 10nM; however, in Fig 4E, cell viability assay doesn't seem to differ at this inhibitor concentration. How do you explain this inconsistency?

Author Response: We apologise if this was unclear. Fig 4D and Fig 4E are addressing two different biological questions. Fig 4E evaluates cellular metabolic activity, while Fig 4D provides an estimate of the growth capabilities of cells that are still alive. The apparent discrepancy is due to the difference in the data collected. Fig 4D describes data obtained

© 2021. Published by The Company of Biologists under the terms of the Creative Commons Attribution License (https://creativecommons.org/licenses/by/4.0/).

by real-time serial sequential measurements of cell growth from cells seeded onto 24-well plates. In contrast, Fig 4E shows data collected as a single end-point measurement of cell metabolic activity from cells grown in a 96-well plate. It is not valid therefore to compare the numbers in these two experiments directly.

8. Reviewer: In Figure 5D, the phosphorylation level of ERK1/2 looks the same between 24 and 72 hr of 10nM Afatinib. If activation of ERK1/2 directly affects mutant aggregates formation, why didn't we see the same effect at 24 hours? Alternatively, if aggregate formation depends on ERK1/2 activation, the level of phospho-ERK1/2
should be significantly different between 24 and 72 hours, but they are not. How do you explain this? Also, quantification of western blots is warranted for better illustration of data and reproducibility.

Author Response: We believe that this time lag before maximal aggregate reduction reflects the time needed for all the biochemical signals and resulting mechanical cues to translate into clearance of the aggregates. Reduction of ERK1/2 activation is probably a trigger for cells to become less activated/ less migratory and to increase cell-cell adhesions. The drastic loss of keratin aggregates at 72h is a consequence of cells becoming more compact with more junction proteins formed to stabilize the keratin filaments, results which are demonstrated in subsequent figures.

We have provided quantification of the western blot shown in Fig 5D, but we do not currently have facilities to repeat this experiment in the present situation.

9. Reviewer: Figure 6D, without proper quantification, the immunofluorescence images are not informative at all. The quality of these images was also relatively low, and hard to assess any differences if any.

Author Response: We have replaced these immunofluorescence images (former Fig 6D, now Supplementary Fig S3G) with the quantitation bar graphs (former Supplementary Fig. S4E, now Fig 6F).

10. Reviewer: Fig 6F-it's hard to assess changes in the number of aggregate formation between 72 hr Afatinib and after washout with +EGF at this magnification (same for Figure 7B, 7C). It would be helpful to include higher magnification of images and quantification either with the imageJ algorithm or count manually. Also, the GFP signal in 72 Afatinib was overexposed, the quality of the images is low.

Author Response: Thank you for these constructive suggestions. We have now included magnified images from regions-of-interest and converted them to grey scale (and use inverse presentation) to improve visibility of immunostained proteins. We have replaced with another representative picture without GFP saturation.
We have provided quantification of images in the form of bar graphs.

11. Reviewer: Fig7A-quality of the desmoplakin western blot was relatively poor compared to the western blot for the same marker in FigS6A.

Author Response: We have replaced with a better quality blot.

Minor edit:
1. Reviewer: "The image-based results were analyzed, and potential hits were identified based on a cut-off of 25% aggregate reduction (from manual counting results), which showed similar readouts when compared against manual counting (Fig. S3B)." -Should be against semi-automated counting?

Author Response: We have modified text in results p5 to explain this more clearly.

<u>Reviewer 2 Comments for the Author:</u>
Major comments:

1) Reviewer: The level of directness between EGFR inhibition and mutant keratin aggregation is unclear. A number of new data elements would be needed to address this.
a. With the exception of the data using the lower dose afatinib for longer time points, the difference in the % of cells displaying reduced mutant K14 aggregates between conditions

© 2021. Published by The Company of Biologists under the terms of the Creative Commons Attribution License (https://creativecommons.org/licenses/by/4.0/).

(Figure 2C, 3D, S3D, 4B) is rather minimal (roughly from 85% to 70%). Is this level of difference expected to be reflected functionally?

Author Response: The dramatic loss of keratin aggregates must be associated with a change in keratinocyte behaviour, as cells take time to become more compact and form stable junctions and filaments. The small difference in % of cells with aggregates in Figures 2C, 3D, S3D, 4B are due to the shorter time of exposure to the compound inhibitors/stimulators.

b. Reviewer: Similar to 1a, the EGF stimulated cells only see increase in aggregation from about 10% to maybe 20% of cells. Is this functionally relevant?

Author Response: The cells were only stimulated with EGF for 3h in this experiment, hence the modest increase in number of cells with aggregates.

c. Reviewer: It seems odd that out of the four drugs used to inhibit EGFR only low dose afatinib seemed to work so well to reduce keratin aggregation. Is there a reasonable explanation for this observation? Afatinib is known to target other ErbB family members, could one of these be involved instead of EGFR?

Author Response: All the FDA-approved inhibitors tested here showed related effects, but most need higher doses. We have added additional experimental data into the text in Results p. 6-7 to address this. We have included pictures and quantification of all 4 drugs in Supplementary figure 4. Most other EGFRi reduce keratin aggregation at higher dose of 100nM after 48h incubation whereas at 10nM they have no effect. We believe that EGFR is the primary target for all these inihibitors.

d. Reviewer: Is there a EGFR specificity prediction for the GW799251X compound? Could it also target other ErbB family members?

Author Response: GW799251X is known to target EGFR and ErbB2,as stated in the main text. We have changed the text to “kinase inhibition targets” for each of the compounds to make it clearer.

2) Reviewer: How is apoptosis involved in the keratin aggregation/disaggregation? The authors state that toxicity is occurring, so presumably, the treated cells treated with the compounds are dying off within a day or so after the 10 micromolar treatment. Given that the cells appear to tolerate the lower dose of afatinib for longer periods of time, is apoptosis not triggered in these low dose afatinib treated cells? and could the keratin aggregation/disaggregation observation simply be tied to the apoptotic program more so than to EGFR-ERK activation?

Author Response: We have not observed any indications that apoptosis plays a role in the processes discussed here. Washout experiments (formerly Fig 6E, now Fig 6G) indicate that the effects of afatinib are largely reversible, which should not be the case if most cells had undergone apoptosis.

3) Reviewer: Quantification is needed throughout the manuscript for all panels with immunofluorescence images. Without this, one cannot draw a convincing conclusion about the data. For instance, having this data in figure 1 would allow one to make conclusions about the directness of the relationship between ERK activation and mutant keratin aggregation.

a. In figure 1B, what percentages of cells display the border localized phospho-ERK1/2 relative to the peripheral aggregated phospho-ERK1/2?

b. Also in figure 1B, since the authors note there is an uneven expression profile of exogenous K14 forms being expressed, is there any correlation between K14 expression (high versus low) and phospho-ERK1/2 localization?

c. In figure 1C, despite all of the cells in the field expressing mutant K14, only two cells display peripheral aggregation pattern for phospho-ERK1/2. Couldn’t one argue then that the majority of mutant K14 expressing cells don’t have activated ERK in the peripheral aggregates?

Author Response: We thank the reviewer for the feedback in point 3 about the lack of clarity around the pERK staining. We agree that there is a lot of variability between cells in the immunofluorescence images of pERK. This may be due to the transient or fluctuating nature of the signal, which is operating in a highly dynamic part of the cells, but we have no way of investigating this for the current paper. We will therefore leave

© 2021. Published by The Company of Biologists under the terms of the Creative Commons Attribution License (https://creativecommons.org/licenses/by/4.0/).

out the immunofluorescence data for pERK from this paper, as this data is not essential. Figure 1 has been reconstructed accordingly.

Minor comments:

1) Reviewer: It isn't clear why the GW799251X compound was chosen above the shortlisted compounds. They all seem to have fairly similar abilities to reduce the % of cells with keratin aggregates (figure S3D).

Author Response: All the hit compounds identified in the kinase inhibitor screen, except maybe one, have targets in or affected by EGF/EGFR signalling, in one or other branch of this multifaceted signalling network. Therefore, we selected GW799251X, or rather its target EGFR, for further investigation because it represents the most upstream target. We have modified the text to try and make this rationale more evident.

2) Reviewer: How do the afatinib concentrations used in the study compare to what is used clinically?

Author Response: This is a very important question and one which we cannot yet answer. As we are unable to directly translate in vitro concentrations to in vivo concentrations, we will need to do this in animal studies first.

3) Reviewer: The "Common et al., manuscript in preparation" citation in the first paragraph of the Results section is unnecessary. There is no allusion to the significance of non-random aggregate distribution in the manuscript, and there is no way to check the data since it is not accessible to the public.

Author Response: Thank you for pointing out that oversight. We have now deleted this reference from the manuscript.

4) Reviewer: There is a Russell 2016 citation with no information. Is this a searchable source?

Author Response: We apologise for this error; we have now corrected the reference in the text and reference list.

5) Reviewer: An observation: the pattern of cells with reduced aggregation appear to be in small clusters in contact with one another (Fig 2, for example).. Might this be an indication of a "seeding" event and/or cell-cell communication that triggers a cascade throughout the field?

Author Response: Yes, that is a very good point which is currently the object of another manuscript.

<u>Reviewer 3 Comments for the Author:</u>

Reviewer: The submission represents an important contribution to our understanding and potential treatment of the skinfragility disease Epidermolysis bullies simplex (EBS). A novel drug screening platform and ImageJ plugin are described and its utility demonstrated by the identification of Afatinib, an EGFR inhibitor and the EGFR signalling pathway in general, to treat EBS.

The experimental system relies on a well-established keratinocyte cell line expressing K14 and the R125P mutant linked to EBS (R125P) and new cell lines based upon N/TERT cells stably transfected with GFP-K14WT and GFP-N14 R125P. This mutation is associated with the Dowling-Meara form of the disease. These KEB7 cells have been examined previously for their biomechanics properties and were shown to be just as capable of surviving large-scale uniaxial strains as cells expressing wild type K14 (Beriault et al 2012), whilst Lulevich et al. (2010) found that KEB7 cells were more compliant and weaker than WT cells when subject to an AFM analysis. It is important to distinguish between apical compression and lateral tension when monitoring the consequences and yet here the group have used a different assay to monitor impact - the resilience of the KEB7 treated cells to a dispase-based dissociation assay. This to me is the weakest part of the submission.

Author Response: There has not yet been a formal demonstration that either shear or compression forces are involved in causing EBS blisters, although it seems highly likely that both are involved at the tissue level. Neither unidirectional stretching nor AFM

© 2021. Published by The Company of Biologists under the terms of the Creative Commons Attribution License (https://creativecommons.org/licenses/by/4.0/).

localised compression would alone mimic the forces impacting the basal cell layer in skin. In view of the known effect of EGF on desmosomes and the known desmosome abnormalilites of EBS cells, we used a different assay here because we are now trying to measure the biological impact of the increased cell-cell junctions resulting from EGFR inhibition, as the increased stress resilience of the epithelial sheet imparted by more desmosomes and other cell cohesion mechanisms. The dispase assay is one favoured by many groups looking at desmosome functionality.

Reviewer: The group had already established that stress kinase pathway, Erk1/2 activation and keratin aggregates are linked. EGF is a known regulator of keratinocyte differentiations and K10 expression. The novelty of this submission is that these different facets have been convincingly connected and set the stage to screen for
EGFR signalling pathway inhibitors. The question remains whether this will be effective in animal models and patients, but at least here there is reason to start along that path.
The manuscript is well written, the data presented in a clear and easily tracked manner.

Author Response: We thank the reviewer for their thoughtful and encouraging comments.

Second decision letter

MS ID#: JOCES/2021/258409

MS TITLE: Scalable assay-identified EGFR inhibition lowers stress activation and increases resilience of EBS keratinocytes

AUTHORS: Tong San Tan, John E. A. Common, John S. Y. Lim, Cedric Badowski, Muhammad Jasrie Firdaus, Steven S. Leonardi, and E. Birgitte Lane
ARTICLE TYPE: Research Article

I am happy to tell you that your manuscript has been accepted for publication in Journal of Cell Science, pending standard ethics checks. Please note the comment about reconsidering the title, and let us know if you would like to make changes, as soon as possible.

Reviewer 1

Advance summary and potential significance to field

This paper investigated the molecular mechanisms underlying the stress-induced misfolded protein response in Epidermolysis bullosa simplex (EBS), a rare skin condition caused by keratin mutations. The authors developed a semi-automated system to quantify intracellular keratin aggregates using human keratinocytes EBS reporter cells stably expressed GFP-tagged EBS-mimetic mutant keratin. Using this tool, the authors screened a library of kinase inhibitor compounds. They found EGF inhibitor to attenuate mutant K14 protein aggregation formation by downregulating ERK1/2 activation, causing the cells to undergo early differentiation,
become quiescent, and improve mechanical resilience cell-cell connectivity and formation of stable filament networks.

Comments for the author

The reviewers have responded to most of the comments/criticisms raised in the initial review. The data provided support the central hypothesis and conclusions.
The authors are encouraged to re-think the title, in its present form it's quite wordy and a bit confusing:
Scalable assay-identified EGFR inhibition lowers stress activation and increases resilience of EBS keratinocytes
Reviewer 2

© 2021. Published by The Company of Biologists under the terms of the Creative Commons Attribution License (https://creativecommons.org/licenses/by/4.0/).

Advance summary and potential significance to field

In this resubmission, the authors have done a great job to address the comments from the first submission. The inclusion of new higher quality microscopy images and quantitation of images and immunoblots throughout are welcomed additions that provided clarity to the data, and they ultimately supported the authors' conclusions that inhibition of EGFR signaling can attenuate the adverse effects on keratinocyte biology caused by mutant keratin aggregation.

The changes made to the text are also welcomed and certainly clarify the authors' rationale (ie. why afatanib was chosen over the other drugs).

The authors were wise to remove the phospho-ERK data from the original figure 1, as the suggestive link between keratin aggregation and ERK1/2 activation is likely to be indirect and, perhaps, they are several steps away from each other. I appreciate that this notion was also highlighted in the first paragraph of the discussion in this resubmission.

Overall, the revised manuscript is a more focused, more polished product and I support its acceptance for publication in JCS. Given that EGFR inhibitors are already applied clinically, it will be fascinating to discover whether EGFR blockade provides a therapeutic benefit to EBS patients.

Comments for the author

No additional criticisms.

Reviewer 3

Advance summary and potential significance to field

My concerns have been allayed. The responses given to the helpful comments made by the other referees have greatly improved the clarity and robustness of the manuscript and the data presented. This will be a valuable tool for the community and beyond to assess protein aggregation and search for small molecules/drugs to prevent their occurrence.

Comments for the author

No further revision is required.

© 2021. Published by The Company of Biologists under the terms of the Creative Commons Attribution License (https://creativecommons.org/licenses/by/4.0/).
